# A fuzzy gene expression-based computational approach improves breast cancer prognostication

**DOI:** 10.1186/gb-2010-11-2-r18

**Published:** 2010-02-15

**Authors:** Benjamin Haibe-Kains, Christine Desmedt, Françoise Rothé, Martine Piccart, Christos Sotiriou, Gianluca Bontempi

**Affiliations:** 1 Functional Genomics and Translational Research Unit, Medical Oncology Department, Jules Bordet Institute, Boulevard de Waterloo, Brussels, 1000, Belgium; 2 Machine Learning Group, Computer Science Department, Université Libre de Bruxelles, Boulevard du Triomphe, Brussels, 1050, Belgium

## Abstract

Early gene expression studies classified breast tumors into at least three clinically relevant subtypes. Although most current gene signatures are prognostic for estrogen receptor (ER) positive/human epidermal growth factor receptor 2 (HER2) negative breast cancers, few are informative for ER negative/HER2 negative and HER2 positive subtypes. Here we present Gene Expression Prognostic Index Using Subtypes (GENIUS), a fuzzy approach for prognostication that takes into account the molecular heterogeneity of breast cancer. In systematic evaluations, GENIUS significantly outperformed current gene signatures and clinical indices in the global population of patients.

## Abstract 2

A fuzzy computational approach that takes into account several molecular subtypes in order to provide more accurate breast cancer prognosis

## Background

Early gene expression studies [1-6] classify breast cancer into at least three clinically relevant molecular subtypes: basal-like (predominantly estrogen receptor (ER) negative and human epidermal growth factor receptor 2 (HER2) negative), HER2-positive, and luminal-like (ER-positive) tumors. Although this classification has changed the way clinicians perceive the disease, it has been difficult to use the initial microarray-based clustering models in clinical practice. The reason is that these models suffer from the drawbacks of the hierarchical clustering method itself, namely its instability and the difficulty associated with using it for new data [7]. To address these concerns, we recently used model-based clustering to introduce an alternative model able to identify different molecular subtypes [8,9]. We have shown that this model is capable of *fuzzy *classification [10,11]: a patient's tumor belongs simultaneously to each molecular subtype with some probability (degree of membership) in a way that is reproducible and robust because clinically relevant molecular subtypes are identified in several public datasets using different populations of breast cancer patients and different microarray technologies. However, we observe that a significant proportion of tumors are elusive with respect to subtype, their phenotype lying between several molecular subtypes.

During recent years, several research groups have used gene expression profiling technology to develop prognostic signatures (reviewed in [12]). These signatures add prognostic information to commonly used clinico-pathological criteria and consequently may help to reduce the current over-treatment of patients by better identifying those patients who will most benefit from treatment. Given this tremendous clinical potential, two of these signatures are now being evaluated in large clinical trials to confirm their prognostic value [13,14].

We demonstrated in a recent meta-analysis of publicly available gene-expression and clinical data from almost 3,000 breast cancer patients that the majority of these prognostic signatures showed similar performance despite the limited overlap of genes [8,9]. Interestingly, we also observed that the proliferation-related genes drove the performance of these signatures, which were useful in classifying ER+/HER2- patients as being at low or high risk for recurrence, but were less informative for the ER-/HER2- (often referred to as the 'triple-negative' subtype due to absence of estrogen, progesterone and HER2 receptors) and HER2+ subgroups of patients whose tumors are mostly highly proliferative and considered, therefore, to be high risk. In addition, clinico-pathological criteria revealed independent prognostic information, suggesting that both genomic and clinical variables could be combined in a common prognostic decision algorithm.

In short, although these signatures provide prognostic information that supplements the currently used clinico-pathological criteria, there is still room for improvement, since they add only minimal value to triple-negative and HER2-positive disease. In this article, we propose a novel, *fuzzy *computational approach for breast cancer prognostication that makes it possible to combine risk prediction models specific to each molecular breast cancer subtype. We refer to this approach as *fuzzy *since the risk prediction for a patient is computed by considering their tumor to belong simultaneously to each of the breast cancer molecular subtypes with some probability.

## Results

### Development of the risk prediction model GENIUS

**Figure 1:**
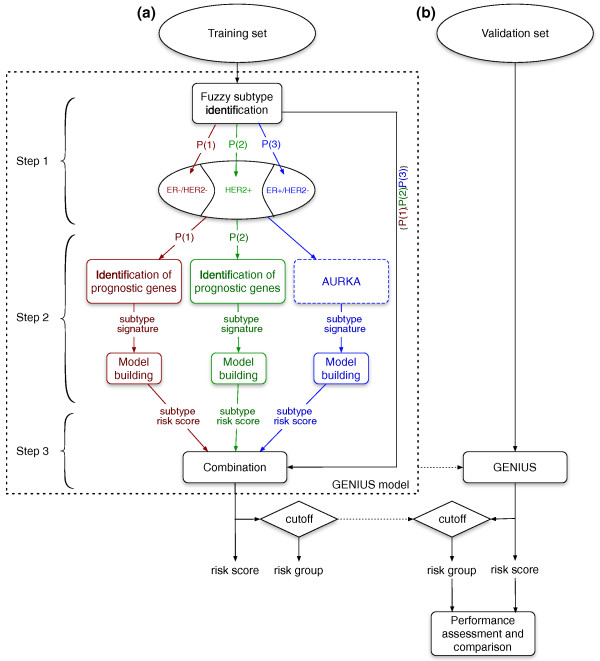
**Risk prediction model design (GENIUS)**. Design of the fuzzy approach used to build the new risk prediction model, called GENIUS (Gene Expression progNostic Index Using Subtypes): **(a) **training phase to build GENIUS; **(b) **validation phase to test GENIUS in the independent dataset of untreated breast cancer patients. For the sake of clarity, we denoted P(ER-/HER2-), P(HER2+) and P(ER+/HER2-) by P(1), P(2) and P(3), respectively.

The novel, fuzzy computational approach we designed for breast cancer prognostication enabled us to build a new risk prediction model, called GENIUS (Gene Expression progNostic Index Using Subtypes). This three-step model is illustrated in Figure 1. Basically, the first step is fuzzy subtype identification by assessing the probability of a patient belonging to each of the three breast cancer molecular subtypes (ER-/HER2-, HER2+ and ER+/HER2-); the second step identifies the prognostic gene signatures specific to each subtype and/or uses existing signatures; and the third step combines the probabilities with the corresponding subtype signature scores, which then results in the final GENIUS risk prediction score. We focused our survival analysis on untreated node-negative patients in order to build a prognostic model for early stage breast cancer and to avoid any confounding factors due to treatment effects on survival (untreated).

#### Identification of the breast cancer molecular subtypes

**Figure 2:**
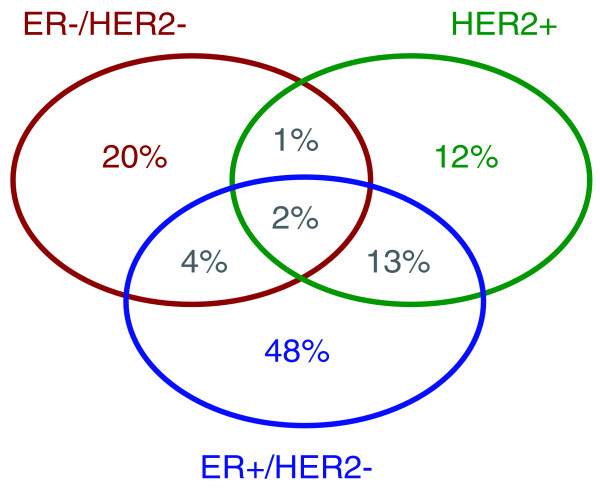
**Proportion of subtypes in primary breast tumors**. Venn diagram of proportions of the three molecular subtypes identified in a database of 3,537 breast cancer patients. We considered a threshold of 1% for the uncertainty of a patient belonging to a specific subtype. Therefore, patients have a tumor of a unique subtype if the posterior probability of belonging to that subtype exceeds 99%.

To assess the probability of a patient belonging to each of the three molecular subtypes, we used model-based clustering in a two-dimensional space [8,9]. These two dimensions were defined by the *ESR1*1 and *ERBB2 *module scores (representing the ER and HER2 phenotypes, respectively), since these genes were shown to be the main discriminators for breast cancer subtyping as confirmed by Kapp *et al*. [2]. In a database of more than 3,300 primary breast tumors retrieved from multiple public datasets (Figure S1 and Table S1 in Additional file 1), we observed a high proportion of well characterized ER+/HER2- subtype (48%) and lower proportions of well characterized ER-/HER2- (20%) and HER2+ (12%) subtypes (Figure 2), which concurs with the literature [15-17]. However, we also found that the tumor subtype for a significant proportion of patients is elusive (Figure 2). For example, we observed that the tumor phenotype lay between the ER+/HER2- and HER2+ molecular subtypes for 13% of the population. The probabilities of patients belonging to each of the breast cancer molecular subtypes are provided in Table S2 in Additional file 1 and Additional file 2.

#### Identification of the subtype prognostic signatures

We used VDX (a breast cancer microarray dataset introduced by Wang, Minn *et al*. [18,19]) as a training set since this population contained the largest sets of ER-/HER2- (99), HER2+ (54) and ER+/HER2- (191) tumors from node-negative patients who had not received any systemic treatment (referred to as 'untreated/').

Many prognostic gene signatures have already been published in the global breast cancer population, and it was shown in a large comprehensive meta-analysis of publicly available expression data that these signatures are informative in the ER+/HER2- subtype and that proliferation-related genes are their common denominator [8]. Given the considerable level of prognostic evidence in this subtype, we did not generate a new prognostic signature for ER+/HER2- tumors, but considered instead the proliferation module (AURKA) [8] as the subtype signature. In contrast, since the ER-/HER2- and HER2+ subtypes represent only small proportions of breast tumors, very few prognostic signatures have been reported thus far for these two subtypes [8,19,20]. Therefore, here we developed a gene selection approach taking into account the probability of a patient belonging to these two subtypes in order to make full use of the available microarray and survival data ('Identification of prognostic genes' in Figure 1 and Additional file 1). We were able to identify two stable signatures composed of 63 and 22 genes for the ER-/HER2- and HER2+ subtypes, respectively (Figure S2 in Additional file 1). The two gene lists selected for each subtype signature are reported in Table S3 in Additional file 1 and in Additional file 3. Their functional analysis is provided in section 4 of Additional file 1.

### Evaluation of the performance of GENIUS

To quantify the risk of relapse of an individual patient, we computed the 'subtype risk scores' for each subtype separately and combined them in a final GENIUS risk score ('Combination'; Figure 1). We then assessed the performance of GENIUS in a validation set, which includes 745 node-negative untreated patients from five publicly available datasets (Table S1 in Additional file 1).

We evaluated the performance of GENIUS in the global population and in the three molecular subtypes in our validation set, the molecular subtype of a patient's tumor being defined by its maximum posterior probability.

#### Risk score predictions

**Figure 3:**
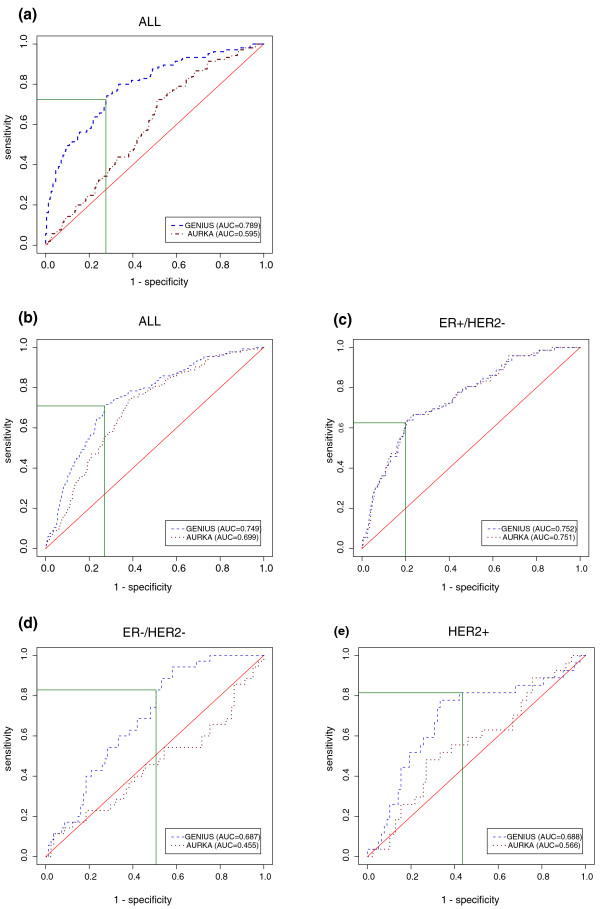
**Time-dependent ROC curves at 5 years for the risk score predictions computed by GENIUS and AURKA**. Training set: in the **(a) **global population of breast cancer patients, to illustrate the cutoff selected for risk group prediction (green lines). Validation set: in the **(b) **global population, the **(c) **ER+/HER2-, **(d) **ER-/HER2- and **(e) **HER2+ subtypes. AUC, area under the curve.

To assess the performance of risk score predictions, we considered the predictions of GENIUS to be continuous scores. We showed that GENIUS was significantly associated with prognosis in the global breast cancer population, as well as in each molecular subtype. In the global population, GENIUS yielded a concordance index (C-index) of 0.71, which may be interpreted as saying that, for any time *t*, the probability was at least 71% that a patient who relapsed at time *t *had a risk score greater than a patient who had not relapsed at time *t*. In the ER+/HER2-, ER-/HER2- and HER2+ subtypes, GENIUS reached a C-index value of 0.70, 0.66 and 0.66, respectively (all *P*-values < 0.001; detailed results are available in Table S4 in Additional file 1). Time-dependent receiver operating characteristic (ROC) curve analysis confirmed these results (Figure 3b-e).

#### Risk group predictions

Risk group predictions (binary variable representing the low- and high-risk groups) were computed by applying a cutoff to the continuous risk scores. Although the categorization of individual risk scores into a small set of risk groups may introduce a bias [21], this approach is intuitive, which must be the case if the risk prediction model is to be used in clinical practice.

The cutoff for the GENIUS risk score was selected so that GENIUS yielded better prognostic performance than the proliferation module (AURKA) in the training set (VDX) using the time-dependent ROC curves (Figure 3a). This choice was made since proliferation-related genes were shown to drive the prognostic value of several prognostic signatures [8,9].

**Figure 4:**
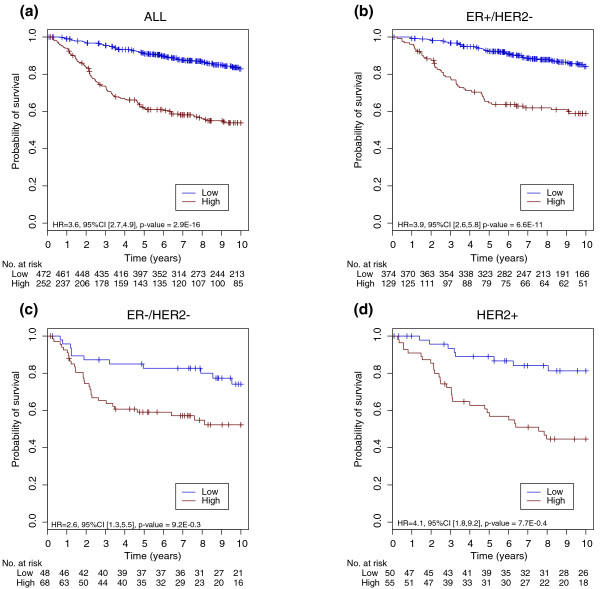
**Survival curves for GENIUS risk group predictions**. Kaplan-Meier survival curves for GENIUS risk group predictions in the **(a) **global population, the **(b) **ER+/HER2-, **(c) **ER-/HER2- and **(d) **HER2+ subtypes of the validation set.

The superiority of GENIUS with the selected cutoff was confirmed in the validation set (Figure 3b-e). We observed a significant difference between the survival curves of low- and high-risk groups predicted by GENIUS for both the global population (hazard ratio 3.7; 95% confidence interval (CI) [2.7,5]; *P *= 1E-16) and all the subtypes: hazard ratios of 3.7 (95% CI [2.5,5.5]; *P *= 1E-10), 2.7 (95% CI [1.3,5.6]; *P *= 7E-3) and 3.9 (95% CI [1.8,8.8]; *P *= 8E-4) in the ER+/HER2-, ER-/HER2- and HER2+ subtypes, respectively (Figure 4). The probability of distant metastasis or relapse free survival of the low-risk group at 5 years was estimated at 91% in the global population, and 92%, 83% and 89% in the ER+/HER2-, ER-/HER2- and HER2+ subtypes, respectively.

As expected, the proportions of patients in the low-risk group differed with respect to the subtypes (Table S5 in Additional file 1). Indeed, we observed lower proportions in the ER-/HER2- (40%) and HER2+ (47%) subtypes than in the ER+/HER2- subtype (74%), these patients being generally at lower risk of relapse.

### Benefit of the fuzzy approach

We sought to further investigate the benefit of the fuzzy computational approach, which assumes that risk prediction can be improved by considering that a patient's tumor belongs simultaneously to each subtype with some probability. Therefore, we developed an alternative risk prediction model - GENIUS CRISP - in order to emphasize this benefit.

The design of GENIUS CRISP is identical to that of GENIUS, except that the probabilities of a patient's belonging to each subtype are not taken into account: a patient is unequivocally assigned to the subtype having the maximum posterior probability (section 7 of Additional file 1). In contrast to the fuzzy approach, this 'crisp' approach is characterized by rough discontinuities at the subtype cluster boundaries, which might introduce undesired effects (increased variance) into the overall risk prediction performance [22,23].

**Figure 5:**
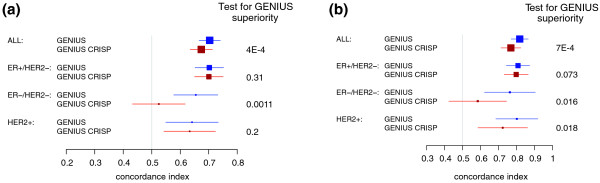
**Forest plot of the concordance indices for GENIUS and GENIUS CRISP**. Forest plot of the concordance indices for GENIUS and GENIUS CRISP risk predictions, with respect to the subtypes in the validation set: **(a) **risk score predictions; **(b) **risk group predictions. The *P*-values at the right-hand side of the forest plot were computed from the statistical test of superiority of GENIUS.

GENIUS CRISP was fitted using the same training set (VDX) as GENIUS. We identified two subtype signatures composed of 10 and 23 genes for the ER-/HER2- and HER2+ subtypes, respectively. Although these subtype signatures were very similar to those identified for GENIUS, up to 15% of the prognostic genes were different in both lists (data not shown). We then computed GENIUS CRSIP risk predictions in our validation set. Although GENIUS and GENIUS CRISP risk scores were highly correlated (0.9 in the global population), GENIUS yielded significantly better performance than GENIUS CRISP, both in the global patient population and in the ER-/HER2- subtype (Figure 5a). The superiority of GENIUS is even clearer for risk group prediction (Figure 5b).

### Comparison with current prognostic gene signatures

Furthermore, in order to determine whether GENIUS would add prognostic information beyond what is provided by already published gene expression signatures, we compared its performance with several signatures shown to be associated with prognosis in the global breast cancer population or in a specific molecular subtype: GGI (gene expression grade index) [24] to represent the initially published prognostic signatures for the global population of breast cancer patients (that is, the GENE70 [25] and GENE76 [19] signatures), since we had previously shown that they all performed similarly [26]; IRMODULE (immune response module) identified by Teschendorff *et al*. [20,27] in the ER-negative breast cancers; SDPP (stroma derived prognostic predictor) representing the stroma-derived prognostic predictor identified by Finak *et al*. [28] and shown to perform well with ER+ and HER2+ tumors; and the *in silico *derived PLAU and STAT1 modules, since our group [8] showed that the immune response module (STAT1) was prognostic in the ER-/HER2- and HER2+ subtypes, while the tumor invasion module (PLAU) was prognostic in the HER2+ subtype only.

#### Risk score predictions

**Figure 6:**
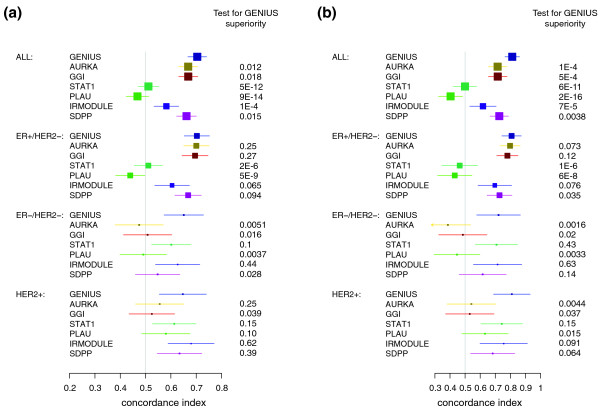
**Forest plot of the concordance indices for GENIUS and the state-of-the-art prognostic signatures**. Forest plot of the concordance indices for GENIUS and the current prognostic signatures (AURKA, GGI, STAT1, PLAU, IRMODULE and SDPP) risk predictions, with respect to the subtypes in the validation set: **(a) **risk score predictions; **(b) **risk group predictions. The *P*-values at the right-hand side of the forest plot were computed from the statistical test of superiority of GENIUS.

GENIUS performed significantly better than all the evaluated gene signatures in the global population of patients (Figure 6a; Table S4 in Additional file 1). However, depending on the signature, the superiority of GENIUS was not always significant in the subtypes in which a particular signature was originally shown to be prognostic. For example, STAT1 and IRMODULE were highly prognostic in the ER-/HER2- and HER2+ subtypes, while SDPP was associated with prognosis in the ER+/HER2- and HER2+ subtypes. We further computed the time-dependent ROC curves at 5 years of the risk score predictions of GENIUS and the existing gene signatures (Figure S5 in Additional file 1) and observed results similar to that of the C-index. The correlation between GENIUS risk score predictions and the current gene signatures are provided in Figure S4 and section 6.1, respectively, in Additional file 1.

#### Risk group predictions

The risk group predictions for the other signatures were computed by applying a cutoff such that the proportions of patients in the low- and high-risk groups were respected as defined by GENIUS. We then compared the performance of GENIUS with the existing gene signatures and observed results similar to that of the risk score predictions (Figure 6b and Table S6 in Additional file 1). Indeed, GENIUS performed significantly better than the other evaluated signatures in the global population of patients. In contrast, in the ER-/HER2- and HER2+ subtypes, STAT1 and IRMODULE were particularly competitive, as was SDPP in the HER2+ subtype only.

In addition to the comparison to individual gene signatures, we sought to further compare GENIUS to SUBCLASSIF, a prognostic model that mimics the use of the best current prognostic gene signatures according to molecular subtype. This crisp risk prediction model is similar to GENIUS CRISP, except that the gene signatures used to compute the subtype risk scores are those already published, that is, the IRMODULE, SDPP and AURKA signatures for the ER-/HER2-, HER2+ and ER+/HER2- subtypes, respectively. It is worth noting that we used different combinations of existing signatures in this framework and obtained similar results (data not shown).

**Figure 7:**
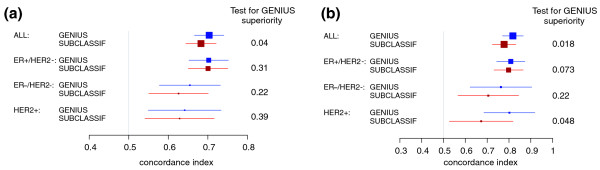
**Forest plot of the concordance indices for GENIUS and SUBCLASSIF**. Forest plot of the concordance indices for GENIUS and GENIUS CRISP risk predictions, with respect to the subtypes in the validation set: **(a) **risk score predictions; **(b) **risk group predictions. The *P*-values at the right-hand side of the forest plot were computed from the statistical test of superiority of GENIUS.

We assessed the performance of SUBCLASSIF in our validation set and observed that it was outperformed by GENIUS, this superiority being significant in the global population of patients for risk score and group prediction (Figures 7a and 7b, respectively). This result suggests that combining novel subtype signatures that take into account the probabilities of belonging to different subtypes yields a better risk prediction model than the one using existing prognostic gene signatures and crisp subtype identification. The correlation between GENIUS and SUBCLASSIF risk score predictions are provided in section 6.1 in Additional file 1.

### Comparison of GENIUS with clinical prognostic indices

In order to evaluate the potential complementarity of GENIUS with the routinely used clinico-pathological parameters, we compared the performance of GENIUS with the Nottingham Prognostic Index (NPI) [29] and Adjuvant! Online (AOL) [30]. We computed NPI risk scores from clinical information, NPI being a simple linear combination of nodal status, histological grade and tumor size. We used the Adjuvant! Online website [31] to compute AOL risk scores.

#### Risk score predictions

The comparison of GENIUS risk scores with those of AOL and NPI yielded correlations of 0.27 and 0.39, respectively, in the global population (Figure S3 in Additional file 1). The correlations were even lower within the ER-/HER2- and HER2+ subtypes. It is worth noting that NPI gave high scores to the great majority of ER-/HER2- and HER2+ tumors.

**Figure 8:**
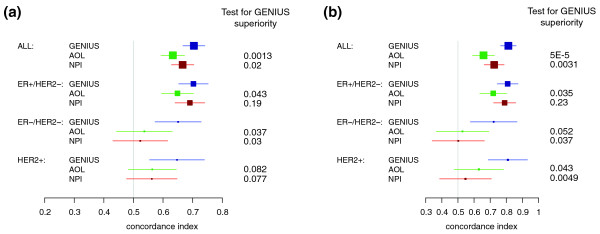
**Forest plot of the concordance indices for GENIUS and the clinical prognostic indices**. Forest plot of the concordance indices for GENIUS and the clinical prognostic indices (AOL and NPI) risk predictions with respect to the subtypes in the validation set: **(a) **risk score predictions; **(b) **risk group predictions. The *P*-values at the right-hand side of the forest plot were computed from the statistical test of superiority of GENIUS.

We also computed the C-indices of AOL and NPI risk score predictions (Table S4 in Additional file 1) and compared them to GENIUS, as shown in Figure 8a. Although GENIUS performed better in the global population, its superiority did not reach significance in all molecular subtypes. In the ER+/HER2- and HER2+ subtypes, for instance, NPI appeared slightly better than GENIUS for high sensitivities, as illustrated in the time-dependent ROC curves at 5 years (Figure S5 in Additional file 1).

#### Risk group predictions

The risk group predictions for AOL and NPI were computed by applying a cutoff that respected the proportions of patients in the low- and high-risk groups as defined by GENIUS. The difference in the survival curves of high- and low-risk patients as defined by AOL and NPI was statistically significant only in the global population and the ER+/HER2- subtype (Figure S6 in Additional file 1). GENIUS significantly outperformed NPI and AOL in the global population of patients and in all subtypes, except for AOL in the ER-/HER2- subtype and NPI in the ER+/HER2- subtype, where GENIUS was not significantly superior (*P*-values for GENIUS superiority of 0.052 and 0.23 respectively; Figure 8b).

### Combination of GENIUS and clinical prognostic indices

**Figure 9:**
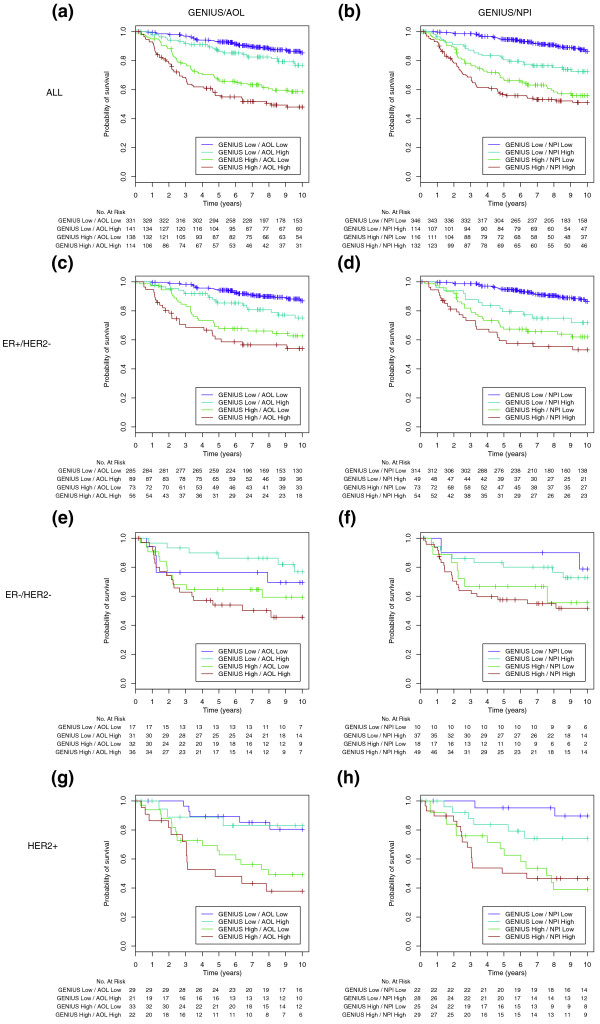
**Survival curves for the combination of GENIUS and the clinical prognostic indices risk group predictions**. Kaplan-Meier survival curves for the combination of GENIUS and AOL/NPI predictions in the **(a) **AOL and **(b) **NPI global population, the **(c) **AOL and **(d) **NPI ER+/HER2-, the **(e) **AOL and **(f) **AOL ER-/HER2- and the **(g) **AOL and **(h) **NPI HER2+ subtypes of the validation set.

The low correlation of the risk score predictions of AOL and NPI with GENIUS raised the question of whether the gene expression and clinical classifiers have complementary value. We therefore drew the Kaplan-Meier survival curves of GENIUS risk group predictions stratified by AOL and NPI classifications (Figure 9). In the global population of breast cancer patients, AOL and NPI seemed to provide additional prognostic information to GENIUS. In the ER+/HER2- subtype, this information seemed to be limited to the patients classified as low-risk by GENIUS. Although we did not observe clear improvement due to the smaller sample sizes of the ER-/HER2- and HER2+ subtypes, AOL and NPI were also correctly able to stratify the patients identified as high-risk patients by GENIUS. Moreover, the combination of GENIUS and NPI seems to be attractive for identifying low-risk HER2+ patients (95% and 90% disease-free at 5 and 10 years, respectively). In order to assess the impact of the cutoff on the combination, we sought to apply the standard cutoffs for NPI [32] and AOL that had been suggested in the TRANSBIG validation studies [33,34]. In these settings, AOL did not add significant information to the ER+/HER2- subtype, whereas NPI exhibited complementarity similar to that observed with the cutoff used for the risk group predictions (Figure S7 in Additional file 1).

### Case studies

**Figure 10:**
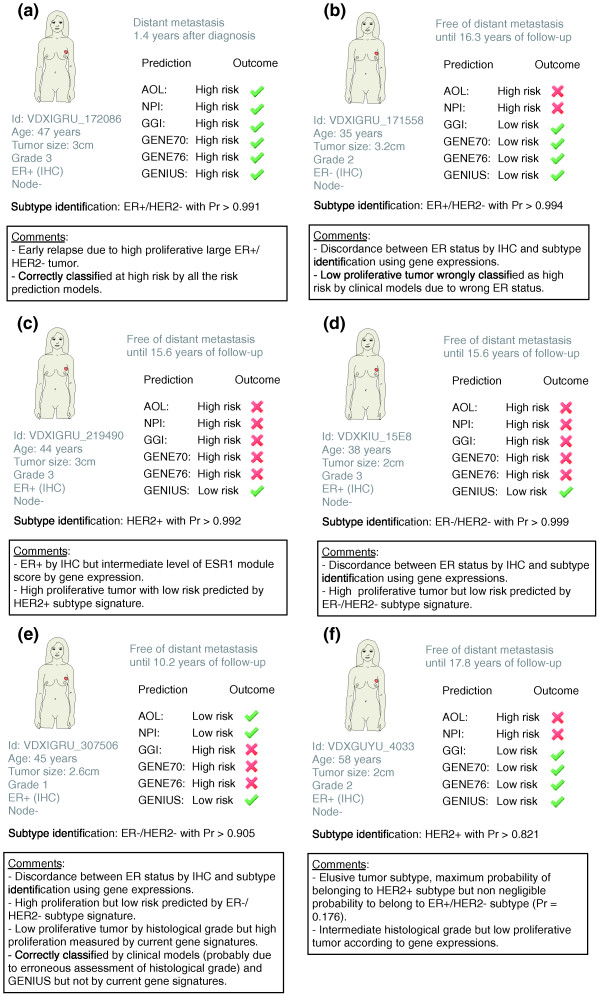
**Study of six breast cancer cases highlighting benefits of using GENIUS**. **(a-f) **Six cases of breast cancer patients from TBG dataset (breast cancer microarray dataset introduced by Desmedt *et al*. [34]) where prognostic clinical indices and gene signatures are compared to GENIUS. The boxes contain relevant comments highlighting the benefits of using GENIUS

**Figure 11:**
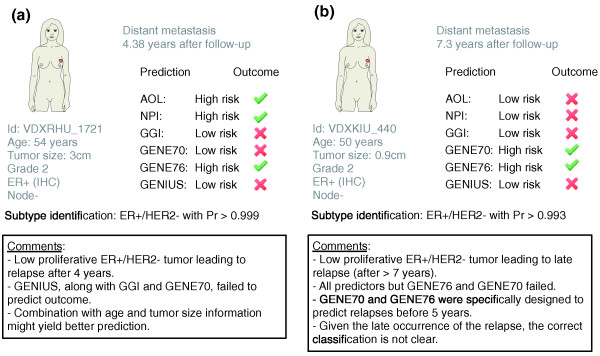
**Study of two breast cancer cases highlighting drawbacks of using GENIUS**. **(a,b) **Two cases of breast cancer patients from the TBG dataset (breast cancer microarray dataset introduced by Desmedt *et al*. [34]) where prognostic clinical indices and gene signatures are compared to GENIUS. The boxes contain relevant comments highlighting the drawbacks of using GENIUS.

In previous sections, we showed that GENIUS significantly outperformed current prognostic gene signatures and clinical indices, especially in the global population of patients. We used the TRANSBIG dataset [34] to illustrate the benefit of using GENIUS when compared to clinical prognostic indices (NPI and AOL) and three official gene signatures (GGI, GENE70, and GENE76). Figures 10a-f and 11a,b describe eight cases of breast cancer with corresponding clinical information and outcome, subtype identification, and official classification computed from prognostic clinical models and gene signatures. Each figure represents a specific case of interest. Figure 10a illustrates the case of a high proliferative large ER+/HER2- tumor correctly classified as high risk by all the risk prediction models. In Figure 10b-f, we illustrate cases that highlight the benefit of using GENIUS over clinical indices and existing gene signatures to identify low-risk breast cancer patients. We observed that GENIUS otperformed clinical indices when there was discordance between ER status assessed by immunohistochemistry and subtype identification using gene expression, especially with elusive tumor subtypes (Figure 10a,b,e10a,b,e). Moreover, for patients whose tumors belonged to the ER-/HER2- and HER2+ subtypes, GENIUS consistently outperformed the prognostic gene signatures (Figure 10a,c,e) and clinical indices in most cases (Figure 10a,c,f). Figures 11a,b represent cases where GENIUS failed to predict clinical outcome. In Figure 11a,b combination of GENIUS and clinical information such as age and tumor size might lead to correct risk assessment for this low proliferative ER+/HER2- tumor, relapsing after 4 years. In Figure 11b, the patient relapsed after 7.3 years (late relapse), making her clinical outcome particularly difficult to predict. These two cases do highlight possible drawbacks from using GENIUS, that is, the absence of age and tumor size information in the model and the potentially poor prediction for late relapses given the different biology for these tumors [35]. Additional comments in Figures 10 and 11 further highlight the potential improvements and drawbacks associated with GENIUS.

## Discussion

In this paper, we introduce a new approach for breast cancer prognostication using gene expression profiling data and taking into account the molecular heterogeneity of breast cancer. This fuzzy computational approach was developed to respond to the major criticism raised with regard to the great majority of gene signatures reported so far, namely that these are only able to identify high- and low-risk patients within ER-positive disease [8,9]. While it is clear that patients with HER2+ and ER-/HER2- breast cancer have an overall prognosis that is worse than that of patients with ER+ disease, some of the former do have a better clinical outcome. However, only few studies have so far attempted to consider the molecular heterogeneity of HER2+ and ER-/HER2- breast cancer and to derive a prognostic predictor for these subtypes [8,20,28].

In 2005, Wang and colleagues [19] were the first to propose the development of a prognostic model by dividing the global population of patients into subgroups based on their ER status. Although the approach seemed appealing and their GENE76 signature performed well, there was still room for improvement. First, the authors considered only two subgroups of patients (ER- and ER+) without taking into account the heterogeneity of HER2+ tumors. Second, the prognostic model specifically developed for ER- tumors was trained on few samples (35) and performed poorly in validation studies [34,36].

In the meta-analyses recently published by our group we observed that the subtype for many breast tumors remains elusive, their phenotype being intermediate between several subtypes. Taking into account this observation, we developed a novel, fuzzy computational approach to build the risk prediction model GENIUS, which is able to determine the prognosis of individual breast cancer patients.

The first step of our approach, the fuzzy subtype identification, consists in assessing the probability that a patient belongs to each of the molecular subtypes (ER-/HER2-, HER2+ or ER+/HER2-). We demonstrated that our two-dimensional clustering model, which considered gene expression modules representing the ER and HER2 phenotypes more precisely than ER and HER2 mRNA levels, was consistently able to identify the different molecular subtypes across 20 publicly available data sets. Although molecular subtype was clearly identified for the majority of patients, one-fifth of patients have elusive tumor subtypes, rendering their cases difficult for risk prediction.

The second step involves identifying prognostic genes through a selection procedure that takes into account the probabilities that a patient belongs to each molecular subtype, and/or uses current gene signatures. We used the proliferation module AURKA for the ER+/HER2- subtype since we had shown previously that this set of proliferation-related genes was highly prognostic in this subtype [8] and was the common denominator of most of published prognostic gene signatures [8,9]. In contrast to the ER+/HER2- subtype, the prognosis of the ER-/HER2- and HER2+ subtypes has been the subject of only few studies, which is why we developed new signatures for these subtypes. Interestingly, our HER2+ subtype signature appeared to be strongly correlated to the immune response modules developed by Teschendorff *et al*. [20] and by our own group [8]. The immune response information contained in this subtype signature was further confirmed by the functional analysis we performed using Ingenuity Pathways. Our ER-/HER2- subtype signature also correlated with the immune response modules [8,20], although to a lesser extent than the HER2+ signature did. These results suggest that studying the immune response mechanisms in these particular subgroups of patients might help us to better understand their tumors and to develop efficient novel targeted therapies.

The third step to our approach consists in combining the probabilities that a patient belongs to each molecular subtype with the corresponding subtype prognostic signature in order to derive a final GENIUS risk prediction score. We showed that GENIUS was highly prognostic in the global population and in all breast cancer subtypes, both when considering GENIUS as a continuous or binary variable. GENIUS was able to identify a significant proportion of low-risk patients within the high-risk breast cancer subtypes ER-/HER2- and HER2+. When we compared GENIUS with SUBCLASSIF, the risk prediction model using the best existing gene signatures according to subtype, we observed that the fuzzy approach used for GENIUS yielded significantly better performance. Moreover, we showed that GENIUS CRISP, the version of GENIUS that is not fuzzy because it does not take into account the probabilities of a patient belonging to each subtype, yielded poorer performance. All of these results strongly support the benefits of our fuzzy approach for breast cancer prognostication. However, although GENIUS was validated in a large retrospective dataset of 745 untreated patients, a randomized clinical trial such as MINDACT [37] would be required to properly evaluate the benefit from using GENIUS in clinical practice.

A criticism raised in recent years with respect to the existing prognostic gene signatures is that they may add little information beyond what is available when using the classic clinico-pathologic parameters according the optimal clinical guidelines. To that end, we considered the NPI and AOL as the references for assessing the risk of recurrence. The prognostic information provided by AOL and NPI then seemed to be limited to the ER+/HER2- subtype. Because we could not compute AOL and NPI on the training set (VDX) due to missing clinical information, we were unable to develop a version of GENIUS fully integrating microarray and clinical data, and to test it on the validation set. However, we observed that the combination of the risk group classification of GENIUS and the clinical guidelines in the validation set might considerably improve the prediction of clinical outcome. Indeed, both AOL and NPI were able to further refine the GENIUS classification in the global population of patients. For the ER+/HER2- subtype, NPI provided a much clearer separation than AOL in the low-risk group of patients, although it takes neither the patient's age nor ER status into account. We might thus hypothesize that within this subgroup of patients with low proliferative tumors, tumor size is the relevant parameter to further refine prognosis in the node-negative breast cancer population. AOL and NPI exhibited only weak prognosis improvement over the GENIUS classification for the ER-/HER2- and HER2+ subtypes. Interestingly, when we did consider the published cutoffs, AOL no longer added significant information to the ER+/HER2- subtype, underlining the importance of the cutoff in evaluating a prognostic indicator.

To further compare GENIUS with prognostic clinical indices and current gene signatures, we illustrated six breast cancer cases retrieved from the TRANSBIG validation study along with the different classifications. We observed that GENIUS was able to identify more low-risk patients, especially when there was discordance between subtypes identified by immunohistochemistry and by gene expression.

Although the GENIUS methodology was used for prognostication in this work, it might be particularly effective to predict response/resistance to anticancer treatments as well. New predictive models using our fuzzy computational approach could be developed by adapting the fuzzy subtype identification step to the biological processes underlying the treatments of interest. Moreover, integrating different sources such as genomic, epigenetic and proteomic data, in addition to transcriptomics, might further improve the performance of the current GENIUS model.

## Conclusions

We report here a novel, fuzzy computational approach to building a risk prediction model to assess breast cancer prognosis that takes into account breast cancer heterogeneity. We have shown that the fuzziness of the approach yielded better performance than a crisp integration of subtype identification and prognostic gene signatures.

## Materials and methods

We developed a fuzzy computational approach to build a new prognostic index for early breast cancer, called GENIUS, which is illustrated in Figure 1. Our method to derive this index is based on a 'divide-and-conquer' strategy, dividing the original problem into simpler ones whose solutions can be combined to obtain a global solution [23]. In this study, the global population of breast cancer patients was divided into fuzzy molecular subtypes for which specific risk prediction models were used and finally combined to get a global risk prediction model. GENIUS was implemented in an R [38] package called genefu, available from the Comprehensive R Archive Network [39].

### Gene expression data

Gene expression datasets were retrieved from public databases or authors' websites: the 20 datasets used in our analysis are described in Table S1 in Additional file 1 and sketched in Figure S1 in Additional file 1. We used normalized data (log2 intensity in single-channel platforms or log2 ratio in dual-channel platforms) as published by the original studies. Hybridization probes were mapped to Entrez GeneID as in Shi *et al*. [40], using RefSeq and Entrez database version 2007.01.21. When multiple probes were mapped to the same GeneID, the one with the highest variance in a particular dataset was selected to represent the GeneID.

### Survival data

For the survival analysis, we considered only node-negative untreated patients (that is, having received neither chemotherapy nor hormone therapy after initial surgical resection with or without radiotherapy). We used distant metastasis free survival as the survival endpoint. However, when distant metastasis free survival was not available (for example, UPP, a breast cancer microarray dataset introduced by Miller *et al*. [41]), we used relapse free survival. We censored the survival data at 10 years in order to have comparable follow-up across the different studies [26,34].

### Fuzzy risk prediction model GENIUS

In this paper we illustrate the methodology employed to develop the risk prediction model GENIUS, which integrates the fuzzy identification of subtypes with novel or existing gene signatures.

#### Fuzzy subtype identification

In order to identify the molecular subtypes of breast cancer, we performed model-based clustering in a two-dimensional space defined by the *ESR1 *and *ERBB2 *module scores, representing the ER and HER2 phenotypes, respectively [8]. Once fitted to the training set, this clustering model returns a set of probabilities of a patient belonging to each cluster (called subtype). These probabilities are denoted by *P*(*s*) where *s *∈ *S *= {ER-/HER2-,GER2+,ER+/HER2-} are the subtypes. We applied this clustering model to several independent datasets to assess its quality and robustness.

#### Identification of prognostic genes

In order to reduce the dimensionality of the gene expression data, we filtered the probes as follows: because we used Affymetrix and Agilent datasets in our survival analysis, we kept only the common genes between these two platforms (10,540 genes); we kept 10% of the genes for which the variance was the largest in the training set.

In order to identify prognostic gene signatures, we used a ranking-based gene selection procedure. The score given to each gene is based on the significance of the concordance index [42] computed by assuming asymptotical normality [43]. We introduced a weighted version of the concordance index in order to select genes relevant for a specific subtype *s*. The weights were defined as the probability of a patient belonging to the subtype *s *(section 3 of Additional file 1).

The only hyperparameter to tune was the signature size *k*, that is, the number of selected genes in the signature. To do so, we assessed the signature stability with respect to its size by re-sampling the training set [44-46].

#### Model building

For a subtype *s*, the subtype risk score, denoted by *R_s_*, was defined as the weighted combination of all the gene expressions in the corresponding signature:

where *Q *is the set of genes in the signature, *n*_*Q *_is the number of genes in *Q*, *x*_*i *_is the expression of gene *i*, and *w*_*i *_is either -1 or +1 depending on its concordance index (*w*_*i *_= 1 if concordance index <0.5, +1 otherwise). Each subtype risk score was scaled such that quantiles 2.5% and 97.5% equaled -1 and +1, respectively. This scaling was robust to outliers and ensured that the risk score lay approximately in [-1,+1], allowing for comparison between datasets using different microarray technology and normalization.

#### Combination

The final risk score for a patient was defined as the weighted combination of the subtype risk scores:

where *P*(*s*) is the probability of belonging to the subtype *s *such that . As the sum of the probabilities equals 1, the final risk score has the same scale as the subtype risk scores. This continuous value quantifies the risk of a patient to relapse, with low and high values denoting low risk and high risk, respectively. We used the final risk score to derive risk groups on the basis of a cutoff defined on the training set.

### Crisp risk prediction model GENIUS CRISP

In order to assess whether the fuzziness of the GENIUS approach improved the overall prognostic ability of the model, we developed a crisp version of GENIUS, called GENIUS CRISP (section 5 of Additional file 1). The design of this risk prediction model is identical to GENIUS except that the probabilities of belonging to each subtype are not taken into account. Indeed, the subtype of each tumor is univocally determined by the maximum posterior probability estimated during the subtype identification step. For instance, the probabilities {P(ER-/HER2-), P(HER2+), P(ER+/HER2-)} = {0.1, 0.8, 0.2} are transformed into {0, 1, 0}.

### Clinical prognostic indices

In order to compare our risk prediction model with the best current clinical prognostic indices, we computed risk predictions using the NPI [32] and AOL version 8.0 [47]. NPI takes into account tumor grade and size and nodal status (the latter being negative for all patients in this study). AOL calculates 10-year survival probability based on a patient's age, tumor size and grade, tumor ER status and nodal status.

### Current prognostic gene signatures

In order to compare our risk prediction model with other gene signatures shown to be prognostic in the global population of breast cancer patients or in specific molecular subtypes, we computed the risk predictions of these signatures using the alternative computational method introduced in Desmedt *et al*. [8]. Although this method may differ from the algorithms used in the original publications, it is able to yield similar performance [8]. Moreover, the strategy used to build our new prediction model (Figure 1) makes it possible to plug these signatures into the subtype signatures in order to assess their potential benefit at the level of the whole model.

### Crisp risk prediction model SUBCLASSIF

In order to mimic the use of the best current prognostic gene signatures according to molecular subtype, we developed a crisp risk prediction model, similar to GENIUS CRISP, except that the gene signatures used to compute the subtype risk scores are those already published. This risk prediction model, called SUBCLASSIF (section 6 of Additional file 1), used the IRMODULE, SDPP and AURKA signatures for the ER-/HER2-, HER2+ and ER+/HER2- subtypes, respectively.

### Performance assessment and comparison

We assessed the performance of the risk score predictions (continuous variable) using the concordance index (C-index) [42], the time-dependent ROC curve [48] and its corresponding area under the curve as implemented in the R package survcomp [49]. The performance of the risk group predictions (binary variable, low- and high-risk groups) was assessed using the concordance index and the hazard ratio estimated through Cox's model. All Cox's models were stratified by dataset, allowing for different baseline hazard functions between cohorts. We statistically compared the performance of the risk score and risk group predictions through C-index by using a paired Student *t*-test [26,50].

### Gene ontology and functional analysis

Gene ontology analyses were performed using Ingenuity Pathways Analysis tools [51], a web-delivered application that enables researchers to discover, visualize, and explore molecular interaction networks in gene expression data. For a more detailed description of the methods, see Additional file 1.

## Abbreviations

AOL: Adjuvant! Online; AURKA: proliferation gene module with prototype AURKA; CI: confidence interval; C-index: concordance index; ER: estrogen receptor; *ERBB2*: gene name for human epidermal growth factor receptor 2; *ESR1*: gene name for estrogen receptor alpha 1; GENIUS: Gene Expression progNostic Index Using Subtypes; GGI: gene expression grade index; HER: human epidermal growth factor receptor; IRMODULE: immune response module; NPI: Nottingham Prognostic Index; ROC: receiver operating characteristic; SDPP: stroma derived prognostic predictor.

## Competing interests

CS, CD, BHK are named inventors of on a patent application for the STAT1 and PLAU modules. CS and MP are named inventors on a patent application for the GGI used in this study. There are no other conflicts of interest.

## Authors' contributions

BHK, CD and GB were responsible for the design and execution of the study, data and statistical analysis and interpretation. FR participated in the data analysis. BHK and CD were responsible for writing the manuscript; MP, GB and CS supervised the study. All authors read and approved the final manuscript.

## Supplementary Material

Additional file 1 Supplementary information, including sections about the identification of molecular subtypes, performance assessment, fuzzy identification of prognostic genes, gene ontology and functional analysis, development of SUBCLASSIF and comparison with GENIUS, development of GENIUS CRISP and comparison with GENIUS, and GENIUS risk predictions for treated patients. The file also contains the supplementary figures and tables. Click here for file

Additional file 2 Comma separated values (csv) file including the probabilities of all the patients belonging to each breast cancer molecular subtypes. Click here for file

Additional file 3 Comma separated values (csv) file including the lists of prognostic genes selected for the subtype signatures. Click here for file
