# Supplementary material for: A fuzzy gene expression-based computational approach improves breast cancer prognostication
Source: Genome Biol. 2010 Feb 15;11(2):R18. doi: 10.1186/gb-2010-11-2-r18 (PMC2872878; doi:10.1186/gb-2010-11-2-r18)
Supplement: Additional file 1 — Supplementary information, including sections about the identification of molecular subtypes, performance assessment, fuzzy identification of prognostic genes, gene ontology and functional analysis, development of SUBCLASSIF and comparison with GENIUS, development of GENIUS CRISP and comparison with GENIUS, and GENIUS risk predictions for treated patients. The file also contains the supplementary figures and tables. [file gb-2010-11-2-r18-S1.pdf]

Supplementary Information

A fuzzy gene expression-based computational approach improves breast cancer prognostication

Benjamin Haibe-Kains[†], Christine Desmedt[†], Francoise Rothé, Martine Piccart, Christos Sotiriou, Gianluca Bontempi

Contents

1 Fuzzy Identification of Molecular Subtypes **3**
1.1 Mixture Modeling 3

2 Performance Assessment **11**

3 Identification of Prognostic Genes **13**
3.1 Weighted Concordance Index 13
3.2 Signature Stability 13

4 Gene Ontology and Functional Analysis **15**
4.1 Pathway Analysis 15
4.2 Correlations with Published Gene Signatures 19

5 Development of GENIUS CRISP and Comparison with GENIUS **20**
5.1 GENIUS vs GENIUS CRISP 20
5.1.1 Risk Score Predictions 20
5.1.2 Risk Group Predictions 23
5.2 Conclusions 24

6 Development of SUBCLASSIF and Comparison with GENIUS **27**
6.1 GENIUS vs SUBCLASSIF 28
6.1.1 Risk Score Predictions 28
6.1.2 Risk Group Predictions 31
6.2 Conclusions 31

7 Supplementary Figures **32**

8 Supplementary Tables **43**

1 Fuzzy Identification of Molecular Subtypes

1.1 Mixture Modeling

In order to identify the molecular subtypes of BC tumors, we performed a clustering in a two-dimensional space. The dimensions were defined by the ESR1 and ERBB2 module scores [8]. These scores were scaled such that quantiles 2.5% and 97.5% equal to -1 and +1 respectively. This scaling was robust to outliers and ensured the module scores to lie approximately in $\{-1, +1\}$, allowing for comparison between datasets using different microarray technology and normalization[1].

We used a simple clustering model that is a mixture of Gaussians with equal variance and shape. We selected the most likely number of clusters with respect to the Bayesian information criterion (BIC) We applied a scaling procedure such that the BIC values for one cluster and the maximum value is equal to 0 and 1 respectively. This allowed for comparison of BIC estimates between different datasets.

We demonstrated that our clustering model was robust by validating it on 20 independent datasets (see Supplementary Table 1). The probabilities for a given tumor to belong to each subtype are given in Supplementary Table 2.

Note that an implementation of our method for breast cancer molecular subtype identification is available form the R package `genefu`[2].

Training set Once fitted on the training set (VDX), this model returns a set of probabilities for a patient to belong to each cluster (called subtype).

As we can see in the figure below (left), the BIC estimates increased dramatically until three clusters and reached a plateau afterwards. Therefore, we considered a mixture of three Gaussians since this number of clusters was likely given the data. The scatterplot in the figure below (right) illustrates the application of this model on the training set, each subtype having a different color and symbol.

[1] As mentioned in the main text of the article, we applied the same scaling procedure to the *subtype risk scores*.

[2] `http://cran.r-project.org/web/packages/genefu/`

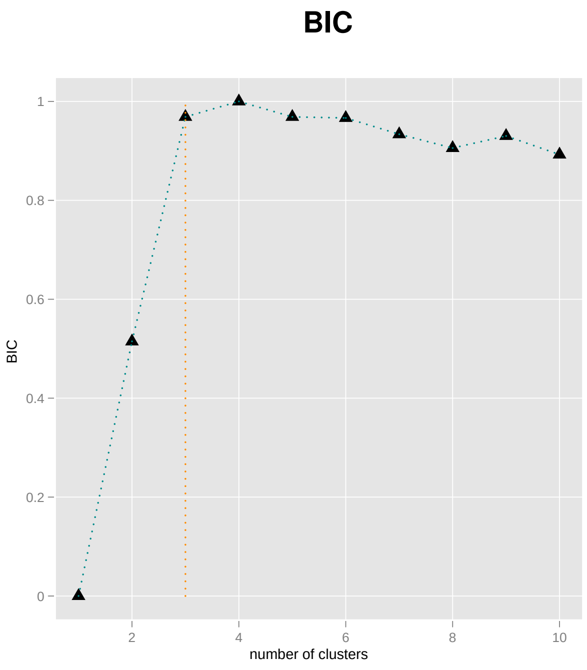

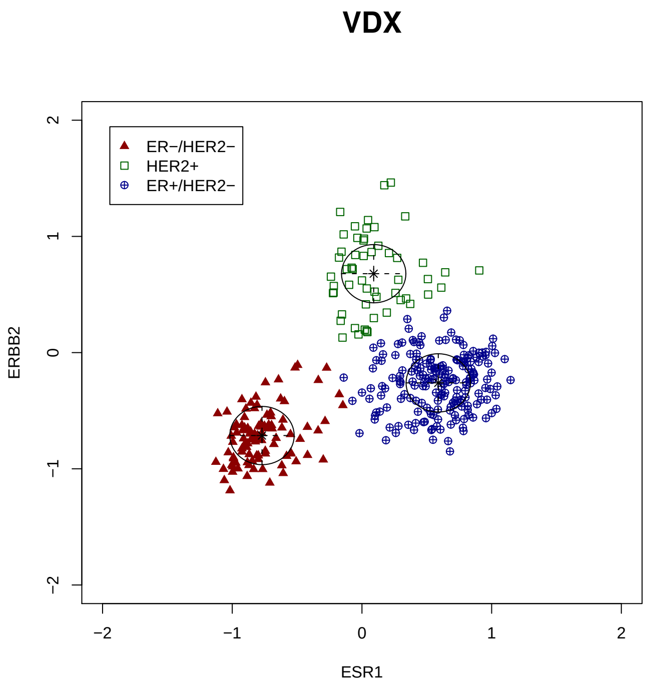

The following table gives the parameters of the clustering model (mixture of three Gaussians with equal shape and variance) as fitted on the training set (VDX).

$\hat{\mu}$	ER-/HER2-	HER2+	ER+/HER2-
ESR1	-0.77	0.09	0.59
ERBB2	-0.71	0.68	-0.26
$\hat{\Sigma} \times I$	0.06	0.06	0.06
$\hat{\pi}$	0.29	0.16	0.56

Independent datasets We applied this model on several independent datasets. Moreover we fitted from scratch a clustering model for each these datasets as for the training set. This allowed for computing the BIC as a function of the number of clusters on each independent datasets to test the goodness of the selected number of clusters. Additionally, we assessed the quality of clustering by computing the prediction strength.

The set of figures below show the identification of the ER-/HER2-, ER+/HER2- and HER2+ molecular subtypes of tumors for each dataset separately. It is worth to note that we were not able to identify the BC subtypes for some publicly available datasets because: (i) the number of probes was not sufficient to compute the ESR1 and ERBB2 module scores; (ii) the BC population of patients was not representative of a global population of breast cancer patients, introducing a bias in the scaling of the ESR1 and ERBB2 module scores (e.g. ER-positive BC cohort).

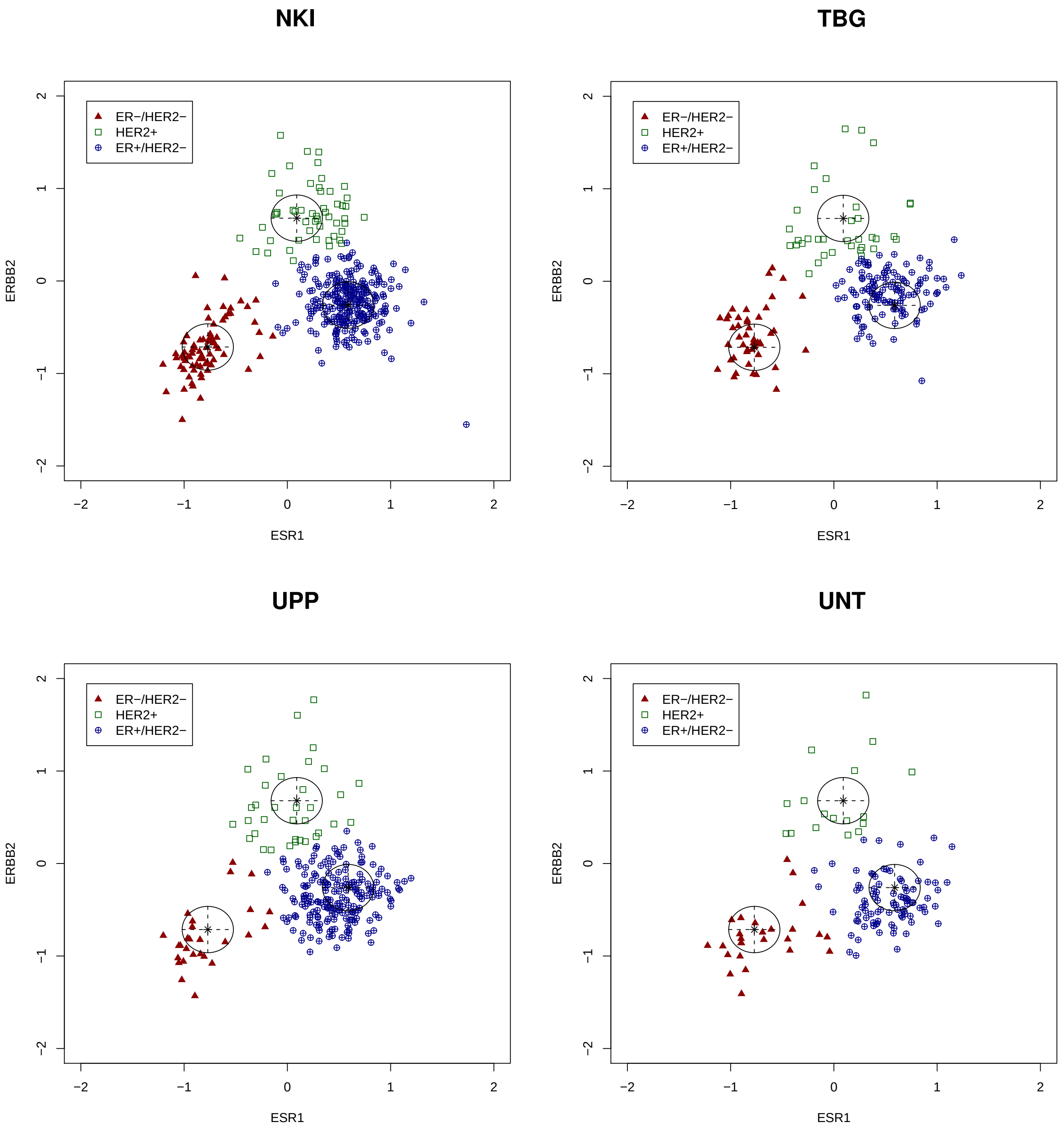
NKI
TBG
UPP
UNT
ER−/HER2−
HER2+
ER+/HER2−
ERBB2
ESR1
−2
−1
0
1
2

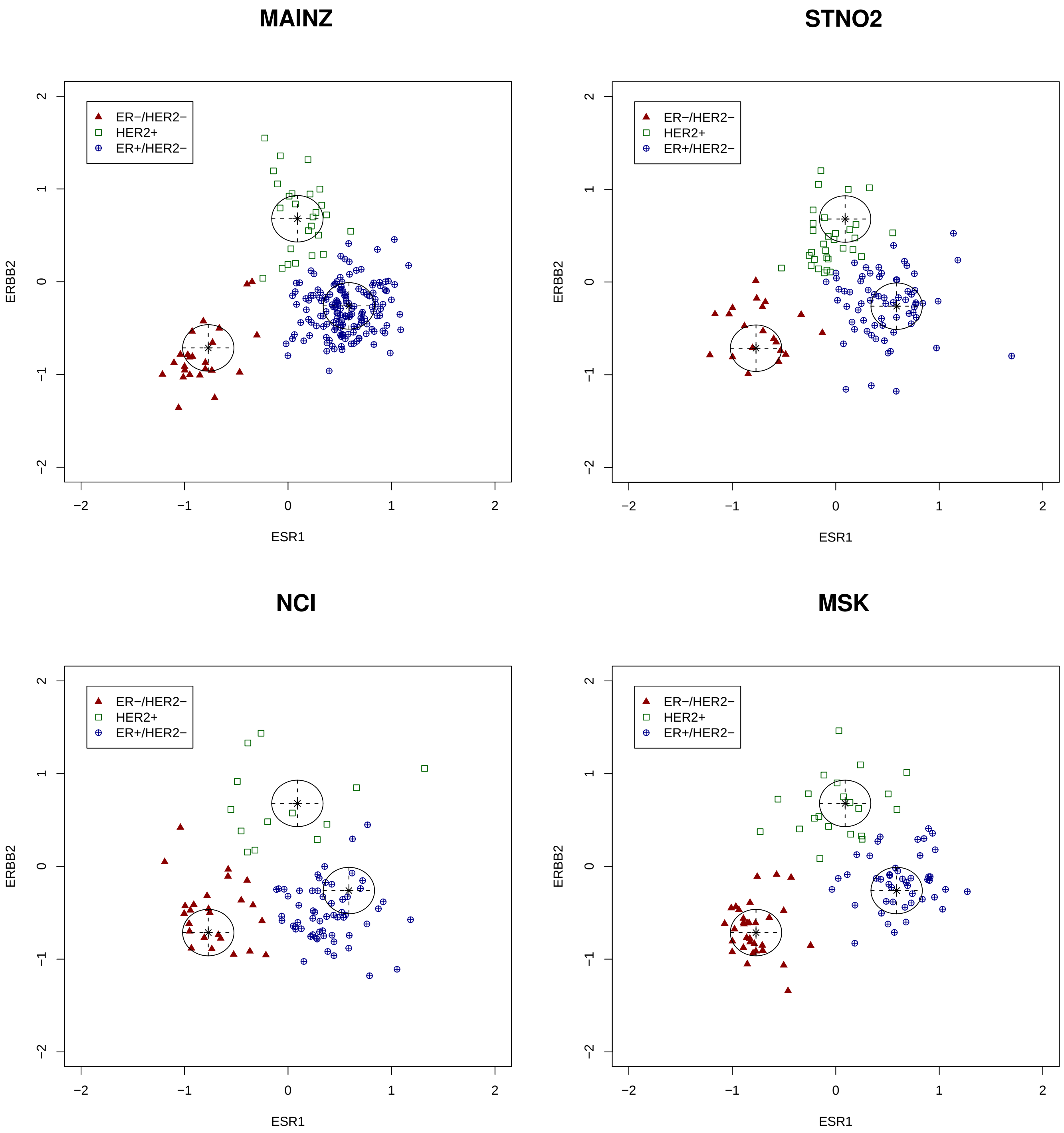
MAINZ
STNO2
NCI
MSK
ER−/HER2−
HER2+
ER+/HER2−
ERBB2
ESR1
−2
−1
0
1
2

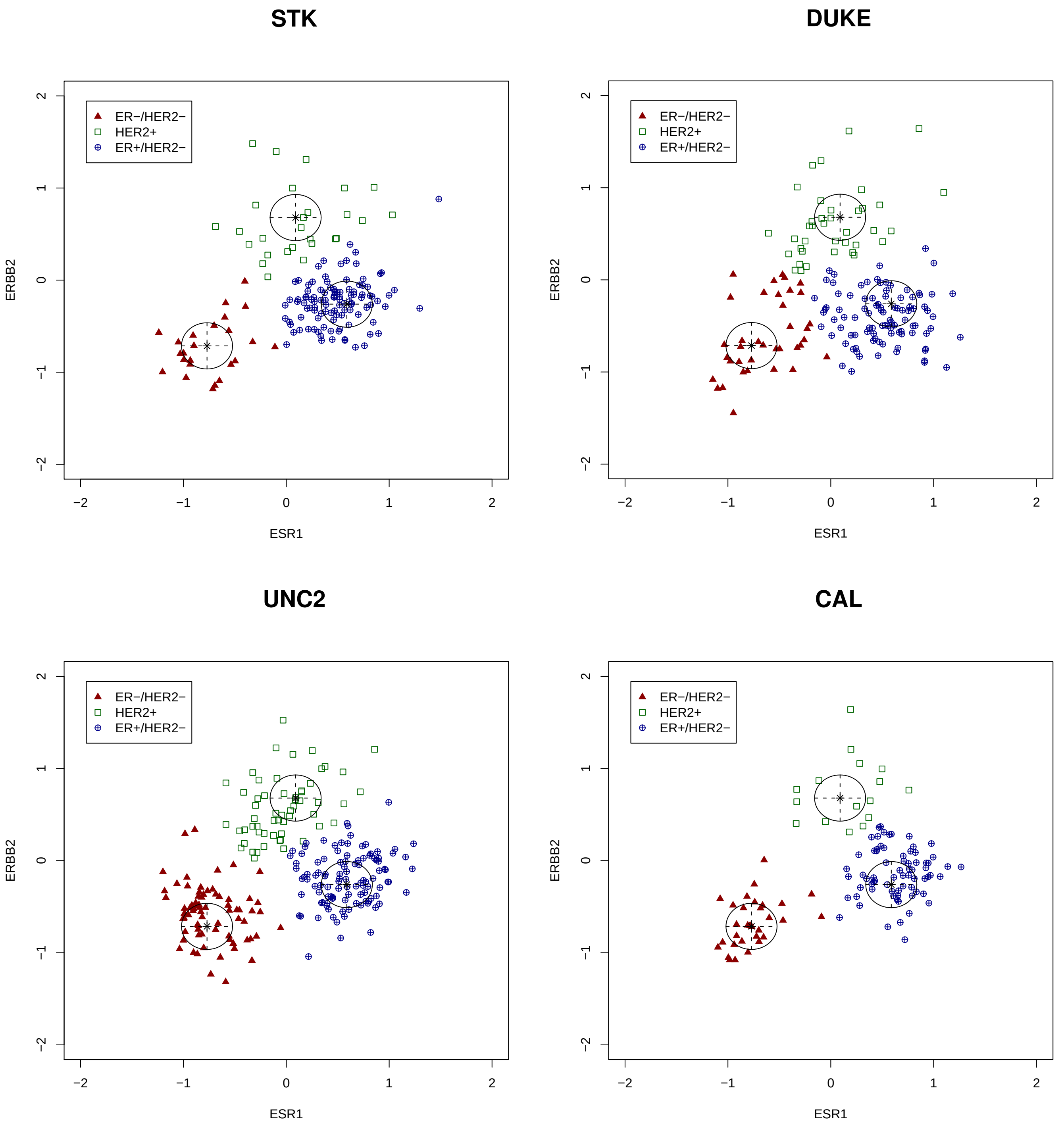
STK
DUKE
UNC2
CAL
ER−/HER2−
HER2+
ER+/HER2−
ESR1
ERBB2

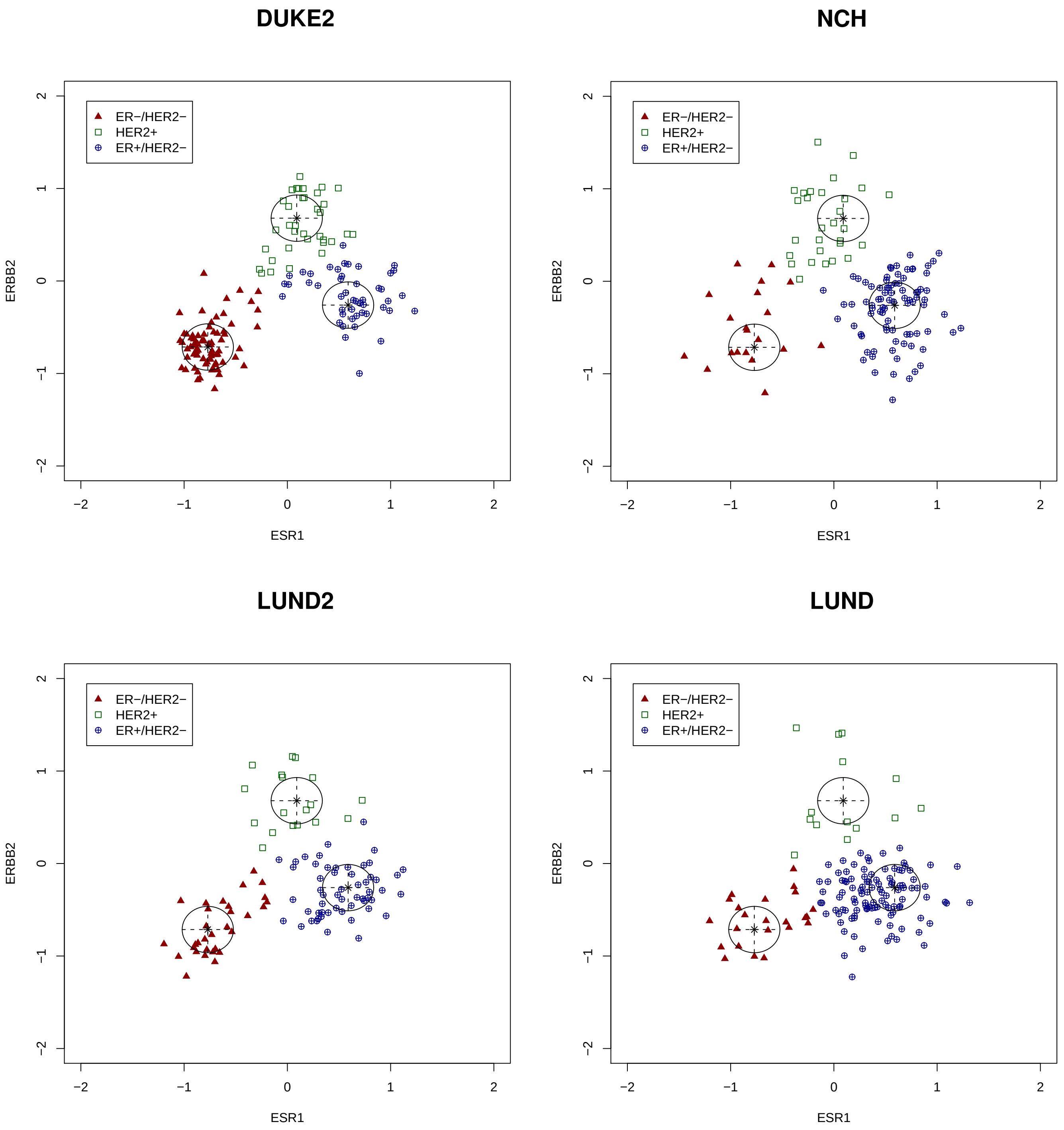
DUKE2
NCH
LUND2
LUND
ER−/HER2−
HER2+
ER+/HER2−
ERBB2
ESR1
−2
−1
0
1
2

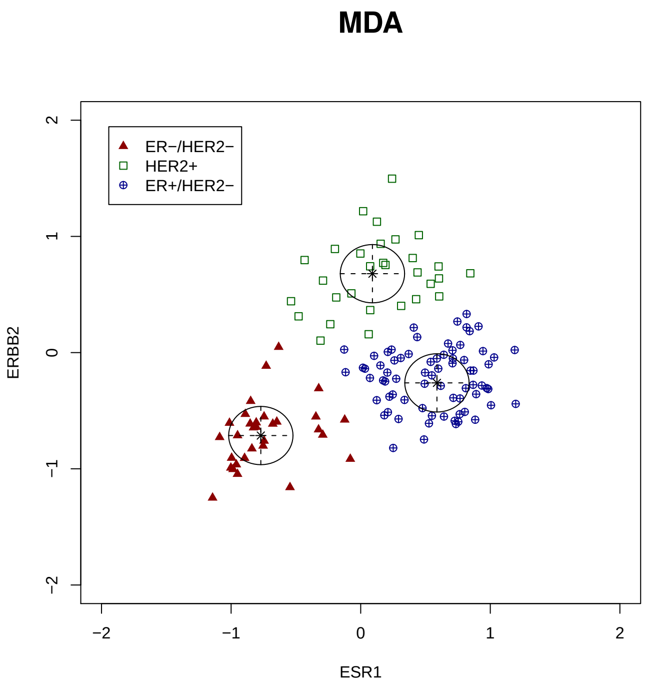

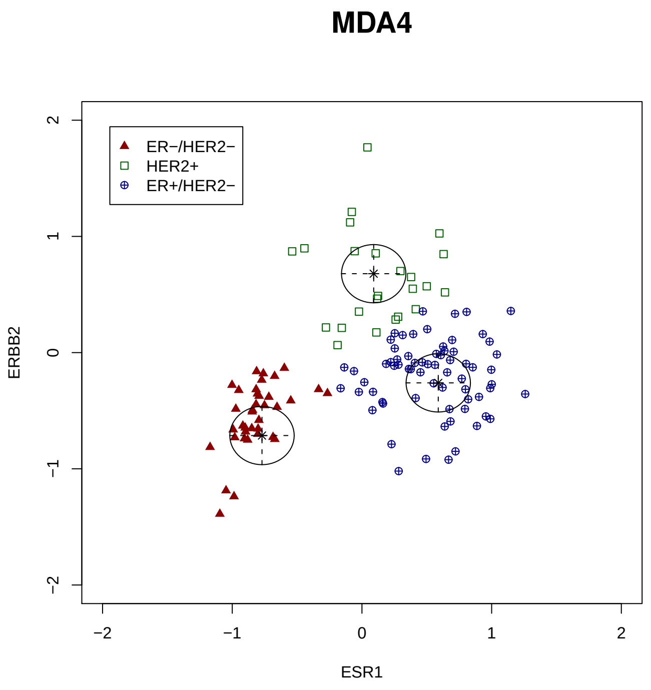

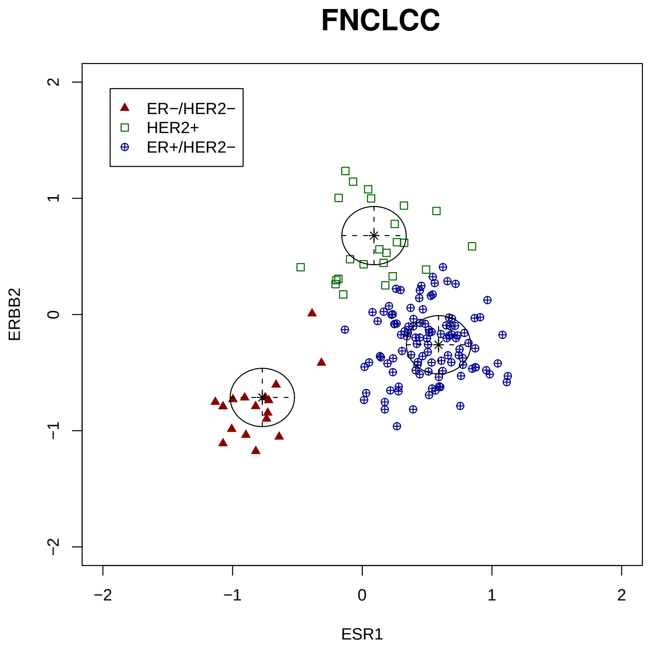

We computed the average of scaled BIC with respect to the number clusters considered in the Gaussian mixture model-based clustering. As sketched by the figure below, we observed that three was the most likely number of clusters, supporting our choice made on the training set.

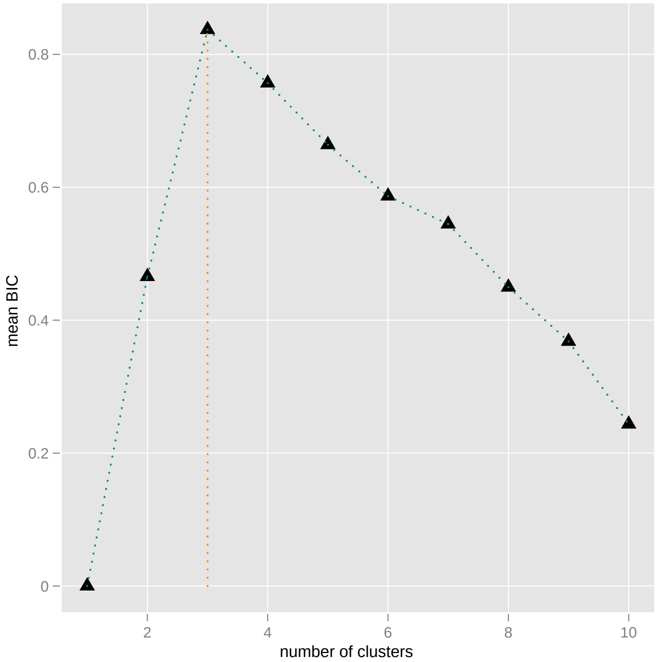

The table below reports the prediction strength of the clustering model with respect to the breast cancer molecular subtypes, in the 20 independent public breast cancer microarray datasets (all datasets but VDX). We observed good prediction strengths ($\geq$ 0.8) for all datasets except for STNO2, DUKE2 and MUG datasets.

Dataset	ER-/HER2-	HER2+	ER+/HER2-
NKI	1.00	1.00	1.00
TBG	1.00	1.00	0.83
UPP	1.00	0.93	0.87
UNT	1.00	0.89	0.92
MAINZ	1.00	1.00	0.90
STNO2	1.00	0.69	0.97
NCI	0.85	0.83	0.93
MSK	1.00	1.00	0.96
STK	1.00	0.91	0.87
DUKE	1.00	0.82	0.92
UNC2	1.00	0.87	0.96
CAL	1.00	1.00	0.95
DUKE2	1.00	0.64	0.95
NCH	1.00	0.82	0.98
LUND2	1.00	0.89	0.87
LUND	1.00	1.00	0.81
MUG	0.66	0.61	0.49
FNCLCC	1.00	0.91	0.93
MDA	1.00	1.00	0.95
MDA4	1.00	1.00	0.73

2 Performance Assessment

We present here the performance criteria used to assess the accuracy of a prognostic gene signature through their risk score and risk group predictions, as implemented in the R package `survcomp`[3]. In the following the quantity r_i and g_i will denote the risk score and the risk group for patient i, respectively. R is a real value and G is either 0 or 1 for a low or high-risk patient respectively.

Let denote the time by t. Survival data for the ith patient are denoted as follows: t_i stands for the event time, c_i for the censoring time, and δ_i for the censoring indicator ($\delta_i = 1$ if $t_i \leq c_i$ and $\delta_i = 0$ if $t_i > c_i$). We introduce the counting process $d_i(t) = 1$ if $t_i \leq t \wedge \delta_i = 1$ and $d_i(t) = 0$ if $t_i > t$ to denote survival status at any time t where $d_i(t) = 1$ indicates that patient i experienced an event prior to time t.

Concordance Index The concordance index (C-index) computes the probability that, for a pair of randomly chosen comparable patients, the patient with the higher risk prediction will experience an event before the lower risk patient. The C-index takes the form

$$C\text{-index} = \frac{\sum_{i,j \in \Omega} 1\{r_i > r_j\}}{|\Omega|}$$

where r_i and r_j stand for the risk predictions of the ith and the jth patient, respectively, and Ω is the set of all the pairs of patients $\{i, j\}$ for whom there is no tie in risk predictions ($r_i \neq r_j$) and who meet one of the following conditions: (i) both patients i and j experienced an event and time $t_i < t_j$ or (ii) only patient i experienced an event and $t_i < c_j$.

Note that the C-index is a generalization of the $AUC(t)$ (with similar interpretation), though it is unable to represent the evolution of performance with respect to time.

Standard Error Standard error, confidence intervals and p-values for the C-index are computed by assuming asymptotic normality.

Time-Dependent ROC Curve The receiver operating characteristic (ROC) curve is a standard technique for assessing the performance of a continuous variable for binary classification. A ROC curve is a plot of sensitivity versus 1 − specificity for all the possible cutoff values of the continuous variable, denoted by c. In survival analysis, the continuous variable is the risk score, and the binary class to predict is the event occurrence, denoted by $D(t)$. As the event occurrence is time-dependent, time-dependent ROC curves are more appropriate than conventional ones. Heagerty et al. proposed to summarize the discrimination potential of a risk score R, estimated at the diagnosis time $t = 0$, by calculating ROC curves for cumulative event occurrence by time t. Once we define the sensitivity SE and the specificity SP as follows

$$\begin{aligned} SE(c, t, r) &= \Pr\{r > c \mid d(t) = 1\} \quad (1) \\ SP(c, t, r) &= \Pr\{r \leq c \mid d(t) = 0\} \quad (2) \end{aligned}$$

[3] `http://cran.r-project.org/web/packages/survcomp/`

the ROC curve $ROC(t)$ at time t is the plot of $SE(c, t, r)$ versus $1 - SP(c, t, r)$ where the cutoff point c is the parameter. In order to estimate the conditional probabilities in (1) and (2), accounting for possible censoring, the nearest neighbor estimator for the bivariate distribution function proposed by Akritas et al. is used preferably to the KM estimator. Indeed the KM estimator does not guarantee that sensitivity and specificity are monotone.

From the time-dependent ROC curve $ROC(t)$ we can summarize the performance of a risk score by deriving the area under the curve quantity, denoted by $AUC(t)$.

$AUC(t)$ lies in $[0, 1]$, the performance of the risk score produced by a random model being equal to 0.5. The performance increases as the departure from 0.5 increases.

Hazard Ratio The hazard ratio can be defined as a summary of the difference between two survival curves, representing the reduction in the risk of event between two different groups. It is a form of relative risk. Proportional hazards regression model assumes that the relative risk of event between the two groups is constant at each interval of time.

Let G be an indicator variable, which takes the value zero if an individual is in the first group (e.g. low-risk group) and unity if an individual is in the second group (e.g. high-risk group). If g_i is the value of G for the ith individual in the study, $i \in \{1, ..., n\}$, the hazard function for this individual can be written as

$$h_i(t) = \lambda_0(t) \exp(\beta g_i)$$

where $g_i = 1$ if the ith individual is on the second condition or zero otherwise. Because of the type of the indicator variable G, $\lambda_0(t)$ is the hazard function for an individual in the first group. Moreover, the hazard function for any individual in the second group is $\psi\lambda_0(t)$ (proportional hazards). ψ is the relative hazard or *hazard ratio* (HR) with $\psi = \exp(\beta)$

This is the proportional hazards model for the comparison of two groups. In this thesis, the indicator variable G is unity for the high-risk group and zero for the low-risk group. So the hazard ratio permits to assess if the risk of the high-risk group is higher than in the low-risk group.

Standard Error Once the parameter β is estimated, giving $\hat{\beta}$, the corresponding estimate of the hazard ratio is $\hat{\psi} = \exp(\hat{\beta})$. The confidence interval of $\hat{\psi}$ can be obtained from the standard error of $\hat{\beta}$. So a $(100 - \alpha)\%$ confidence interval for the true hazard ratio ψ, can be obtained by exponentiating the confidence limit for β because the distribution of the logarithm of the estimated hazard ratio will be more closely approximated by a normal distribution than that of the hazard ratio itself.

3 Identification of Prognostic Genes

We used a fuzzy ranking-based gene selection method to identify the prognostic genes in a specific breast cancer molecular subtype. The score given to each gene is based on the significance of the *weighted* concordance index. We introduced the weighted version of the concordance index in order to select genes relevant for a specific subtype (see Section 3.1), making fuzzy our feature selection method. The weights were defined as the probability for a patient to belong to the subtype of interest.

The only hyperparameter to tune was the signature size k, i.e. the number of selected genes in the signature. To do so, we assessed the stability with respect to the signature size by resampling the training set (see Section 3.2).

3.1 Weighted Concordance Index

Survival data for the ith patient, $i \in \{1, 2, ..., n\}$, are denoted as follows: t_i stands for the event time, c_i for the censoring time, and δ_i for the censoring indicator ($\delta_i = 1$ if $t_i \leq c_i$ and $\delta_i = 0$ if $t_i > c_i$). We introduce the counting process $d_i(t) = 1$ if $t_i \leq t \wedge \delta_i = 1$ and $d_i(t) = 0$ if $t_i > t$ to denote survival status at any time t where $d_i(t) = 1$ indicates that patient i experienced an event prior to time t.

The concordance index (C-index) computes the probability that, for a pair of randomly chosen comparable patients, the patient with the higher risk prediction will experience an event before the lower risk patient. The C-index takes the form

$$C\text{-index} = \frac{\sum_{i,j \in \Omega} 1\{r_i > r_j\}}{|\Omega|}$$

where r_i and r_j stand for the risk predictions of the ith and the jth patient, respectively, and Ω is the set of all the pairs of patients $\{i, j\}$ for whom $r_i \neq r_j$ (no ties in r) and meet one of the following conditions: (i) both patients i and j experienced an event and time $t_i < t_j$ or (ii) only patient i experienced an event and $t_i < c_j$.

We introduced a weighted version of the concordance defined as

$$C\text{-index}_{wted} = \frac{\sum_{i,j \in \Omega} w_{ij} 1\{r_i > r_j\}}{\sum_{i,j \in \Omega} w_{ij}}$$

where $w_{ij} = w_i w_j$ is the weight for the pair of patients $\{i, j\}$. Note that in this study, the weights w_i were defined as the probability to belong to a specific breast cancer molecular subtype (see Figure 1 in the main manuscript).

Standard errors, confidence intervals and p-values for the C-index are computed by assuming asymptotic normality. Note that, in this study, the larger C-index, the better is the predictability of time to event.

3.2 Signature Stability

The selection of the signature size was performed according to a stability criterion that assesses the stability of the ranking for different signature size and selects the most stable size. Let X be the set of features and $freq(x_j)$ be the number of sampling steps in which a feature $x_j \in X$ has been selected out of m sampling steps. The set X is sorted by frequency

into the set $x_{(1)}, x_{(2)}, \ldots, x_{(n)}$ where $freq(x_{(i)}) \geq freq(x_{(j)})$ if $i < j$ where $i, j \in \{1, 2, \ldots, n\}$. A first measure of stability for a given signature size k is returned by

$$Stab(k) = \frac{\sum_{i=1}^{k} freq(x_{(i)})}{km}$$

This statistic is equal to 1 if the same signature is always selected over sampling steps. In the case of no overlap, *Stab* is equal to $\frac{1}{m}$ if $k > 0$ and 0 otherwise. However, since the *Stab* statistic can be made artificially high by simply increasing k, we formulated an adjusted statistic

$$Stab_{adj}(k) = \max\left\{0, Stab(k) - \alpha\frac{k}{n}\right\}$$

where α is a penalty factor depending on the number of selected features. In our study the penalty factor α was fixed to 1 in order to facilitate the selection of the trade-off between signature size and stability. Indeed, the $Stab_{adj}$ criterion is equal to 0 for the two extreme cases, i.e. when either no feature or all ones are selected.

4 Gene Ontology and Functional Analysis

In order to characterize the three subtype signatures, we used two different approaches: Pathway analysis and correlations with published gene expression signatures.

4.1 Pathway Analysis

The signature for the ER-/HER2+ subtype is composed of 63 unique genes. Information was found in the IPKB for 50 of these genes and 37 were significantly associated with a particular function such as cell death (n=21 genes), cellular movement (n=16), immune response (n=11), molecular transport (n=10) and cell-to cell interactions (n=10). There are 22 genes included in the HER2+ subtype signature. Twenty could be used for functional analysis and these genes were significantly associated with the following ontology classes: cancer-related functions (n=11), cellular growth and proliferation (n=9), gene expression (n=7) and immune response (n=6). For the ER+/HER2- subtype, we used our proliferation module (AURKA), which, as reported previously represents mainly cell cycle and proliferation genes.

The following figures report the results from the Ingenuity Pathway Analysis of the subtype signatures for the (a) ER-/HER2-, (b) HER2+ and (c) ER+/HER2- subtypes.

A

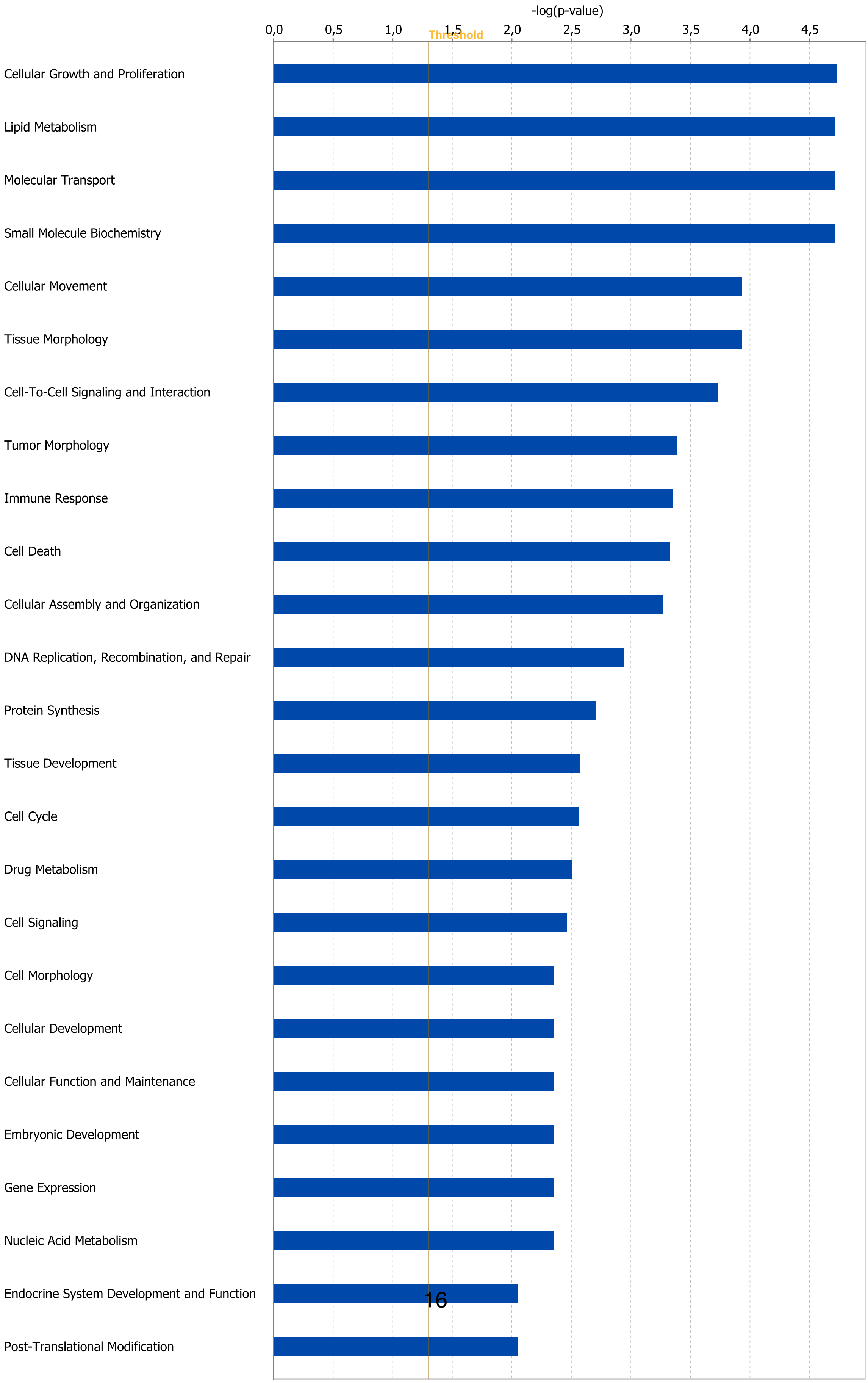
-log(p-value)
0,0
0,5
1,0
1,5
2,0
2,5
3,0
3,5
4,0
4,5
Threshold
Cellular Growth and Proliferation
Lipid Metabolism
Molecular Transport
Small Molecule Biochemistry
Cellular Movement
Tissue Morphology
Cell-To-Cell Signaling and Interaction
Tumor Morphology
Immune Response
Cell Death
Cellular Assembly and Organization
DNA Replication, Recombination, and Repair
Protein Synthesis
Tissue Development
Cell Cycle
Drug Metabolism
Cell Signaling
Cell Morphology
Cellular Development
Cellular Function and Maintenance
Embryonic Development
Gene Expression
Nucleic Acid Metabolism
Endocrine System Development and Function
Post-Translational Modification

B

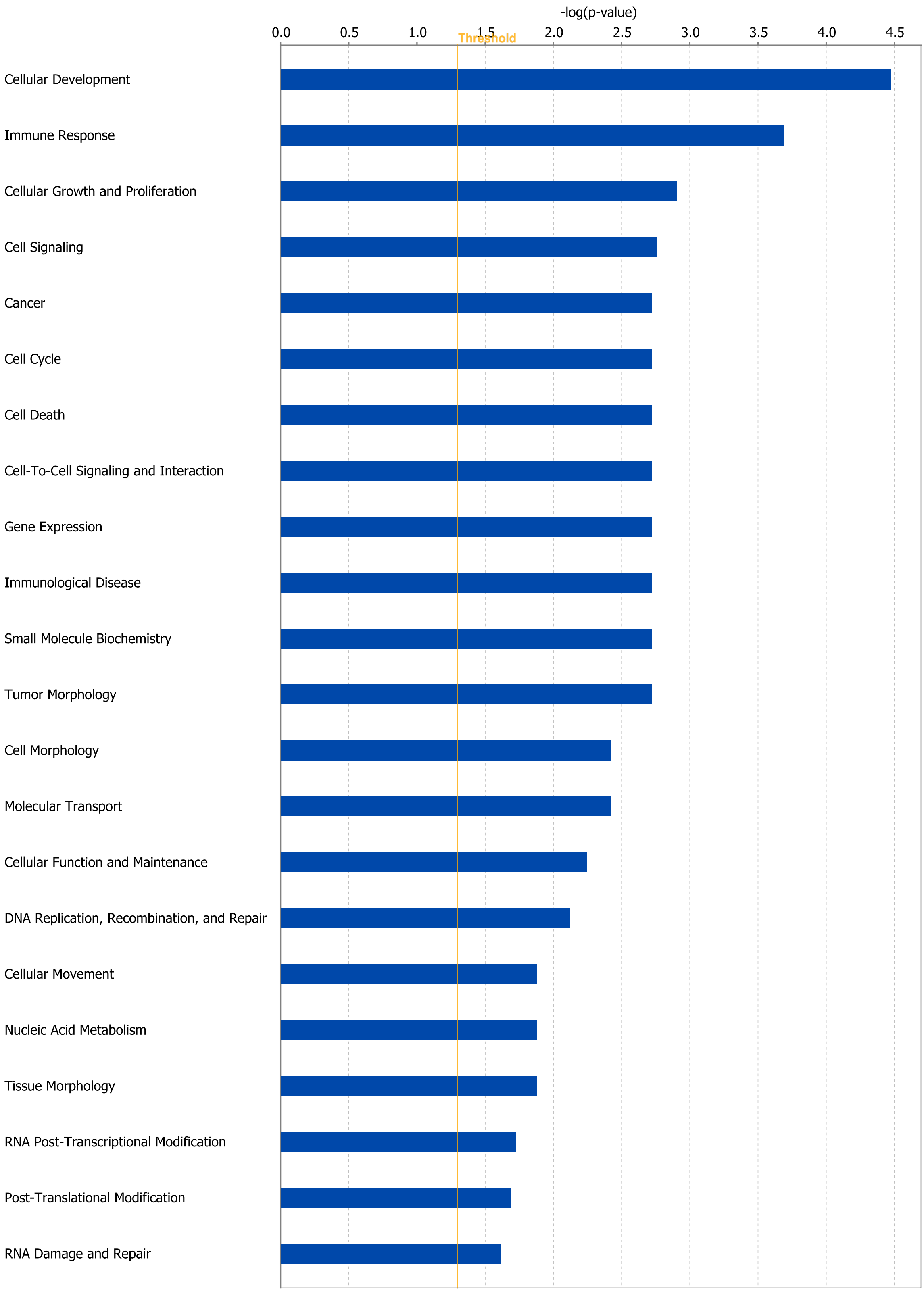
-log(p-value)
0.0
0.5
1.0
1.5
2.0
2.5
3.0
3.5
4.0
4.5
Threshold
Cellular Development
Immune Response
Cellular Growth and Proliferation
Cell Signaling
Cancer
Cell Cycle
Cell Death
Cell-To-Cell Signaling and Interaction
Gene Expression
Immunological Disease
Small Molecule Biochemistry
Tumor Morphology
Cell Morphology
Molecular Transport
Cellular Function and Maintenance
DNA Replication, Recombination, and Repair
Cellular Movement
Nucleic Acid Metabolism
Tissue Morphology
RNA Post-Transcriptional Modification
Post-Translational Modification
RNA Damage and Repair

C

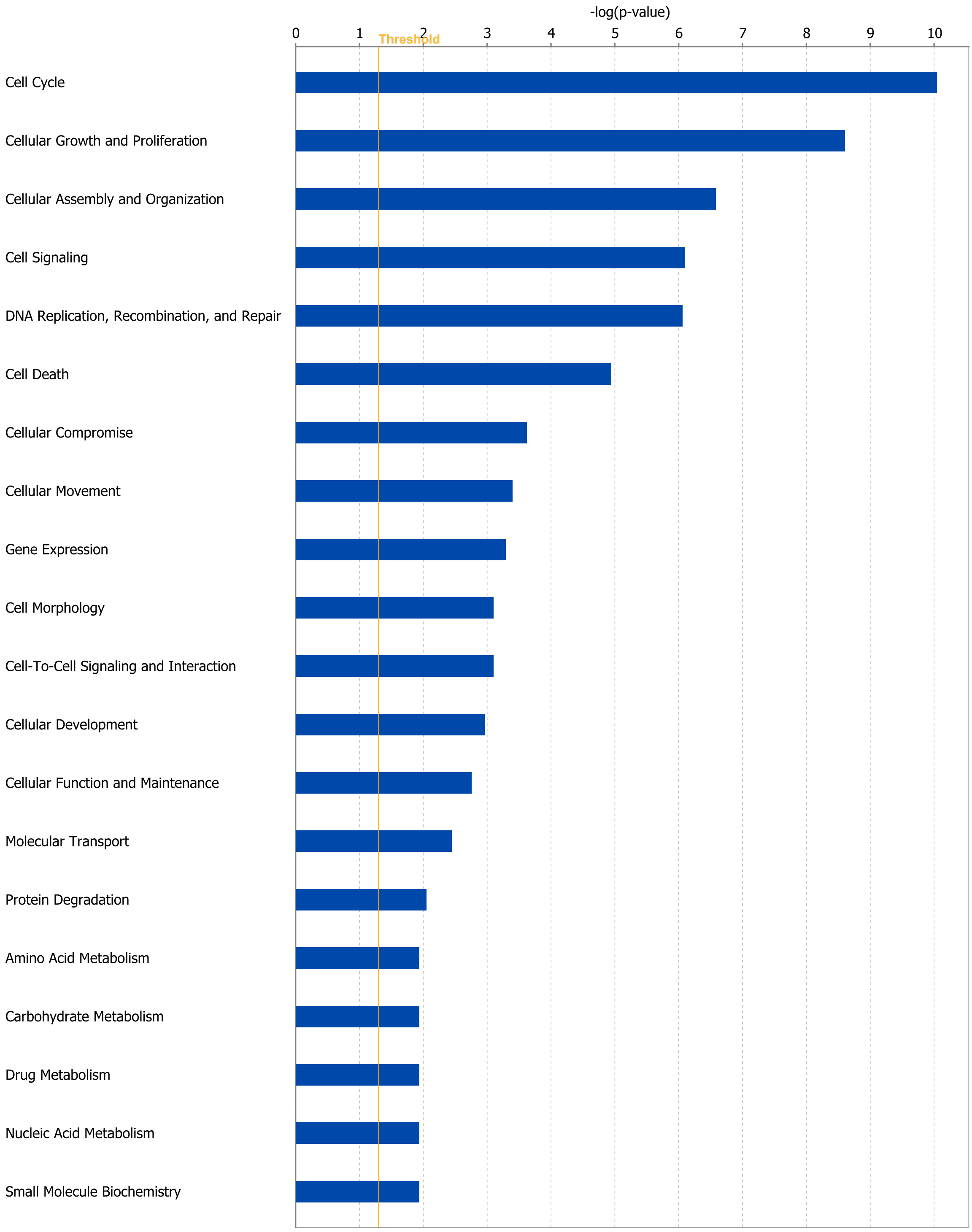
-log(p-value)
0
1
2
3
4
5
6
7
8
9
10
Threshold
Cell Cycle
Cellular Growth and Proliferation
Cellular Assembly and Organization
Cell Signaling
DNA Replication, Recombination, and Repair
Cell Death
Cellular Compromise
Cellular Movement
Gene Expression
Cell Morphology
Cell-To-Cell Signaling and Interaction
Cellular Development
Cellular Function and Maintenance
Molecular Transport
Protein Degradation
Amino Acid Metabolism
Carbohydrate Metabolism
Drug Metabolism
Nucleic Acid Metabolism
Small Molecule Biochemistry

4.2 Correlations with Published Gene Signatures

In order to gain further insight into the biological information included in the subtype signatures, we evaluated their correlation within their respective subtype to gene expression signatures representing known biological processes: ESR1, ERBB2, AURKA, STAT1, PLAU, VEGF, CASP3 modules, IRMODULE , SDPP, and GGI. These correlations were estimated from our database of $\approx$ 3500 primary breast tumors by combining the Fisher's Z transformed correlation coefficients through a meta-analytical framework as implemented in the R package `survcomp`.

As illustrated in the table below, the ER-/HER- signature was significantly associated with the 2 immune response modules within the ER-/HER2- subtype, IRMODULE ad STAT1. Interestingly, the HER2+ signature was even more strongly associated with these immune response modules. In addition, this signature also correlated with our previously described tumor invasion module (PLAU). As expected and as reported previously, the AURKA signature is strongly correlated to the Genomic Grade Index (GGI) within the ER+/HER2- subtype.

	ER-/HER2- signature in ER-/HER2- tumors	HER2+ signature in HER2+ tumors	AURKA signature in ER+/HER2- tumors
ESR1	-0.07	0.18	0.17
ERBB2	0.00	-0.09	0.25
AURKA	-0.18	-0.27	1
STAT1	-0.55	-0.70	0.16
PLAU	0.08	0.48	-0.36
VEGF	0.05	0.29	0.33
CASP3	0.00	-0.01	0.06
IRMODULE	-0.49	-0.67	-0.06
SDPP	-0.24	-0.29	-0.49
GGI	-0.18	-0.24	0.91

Table 7: Pearson correlation coefficients between the current gene signatures (in rows) and the subtype gene signatures used in GENIUS (in columns).

5 Development of GENIUS CRISP and Comparison with GENIUS

In this section, we describe the development of the *crisp* version of GENIUS, called GENIUS CRISP (see Figure 8). In this model, the subtype of each tumor was assigned univocally to the one having the maximum posterior probability estimated during the subtypes identification step (Section 2). Therefore, the probabilities P(1), P(2) and P(3) were not taking into account anymore to identify the *subtype signatures* and to combine the *subtype risk scores* (contrary to GENIUS, see Figure 1 in the paper).

Crisp identification of prognostic genes We used the traditional concordance index to identify prognostic genes in a specific subtype. Contrary to the weighted concordance index used for GENIUS (see Section 3.1), only the tumors belonging to the subtype of interest are used to estimate the concordance index.

The only hyperparameter to tune was the signature size k, i.e. the number of selected genes in the signature. To do so, we assessed the stability with respect to the signature size by resampling the training set (see Section 3.2).

Figure 9 sketches the subtype signature stability with respect to the signature size for the ER-/HER2- and HER2+ subtypes. The number of genes to include in the subtype signature (orange dashed line) was selected in order to maximize the stability with respect to the size by sampling 90% of the training set 200 times. In these settings, 10 and 23 prognostic genes were selected for the ER-/HER2- and HER2+ subtype signatures respectively. Although these subtype signatures were very similar to those identified for GENIUS, up to 15% of the genes were different for both lists (data not shown).

Model building The subtype risk scores are computed as for GENIUS (see *Methods* in the main manuscript).

Crisp decision The final risk score of a tumor is defined as the subtype risk score corresponding to its subtype (see Figure 8). No combination of subtype risk scores is performed.

5.1 GENIUS vs GENIUS CRISP

In order to assess the potential improvement of the fuzzy (GENIUS) over the crisp prognostic model (GENIUS CRISP), we statistically compared their prognostic performance.

5.1.1 Risk Score Predictions

Figure 10 illustrates the GENIUS and GENIUS CRISP risk score predictions with respect to the subtypes. The Pearson's correlation coefficients are given in Table 12. We observed a low correlation within the ER-/HER2- subtype, while the correlation is high for the HER2+ and ER+/HER2- subtypes. Overall, we observed a high correlation between GENIUS and GENIUS CRISP risk score predictions as expected given the large maximum posterior probabilities for the vast majority of tumors in the training and validation sets (see Supplementary Table 2).

In order to assess whether the differences observed at the risk score predictions level have any impact on prognosis, we compared the performance of GENIUS and GENIUS CRISP.

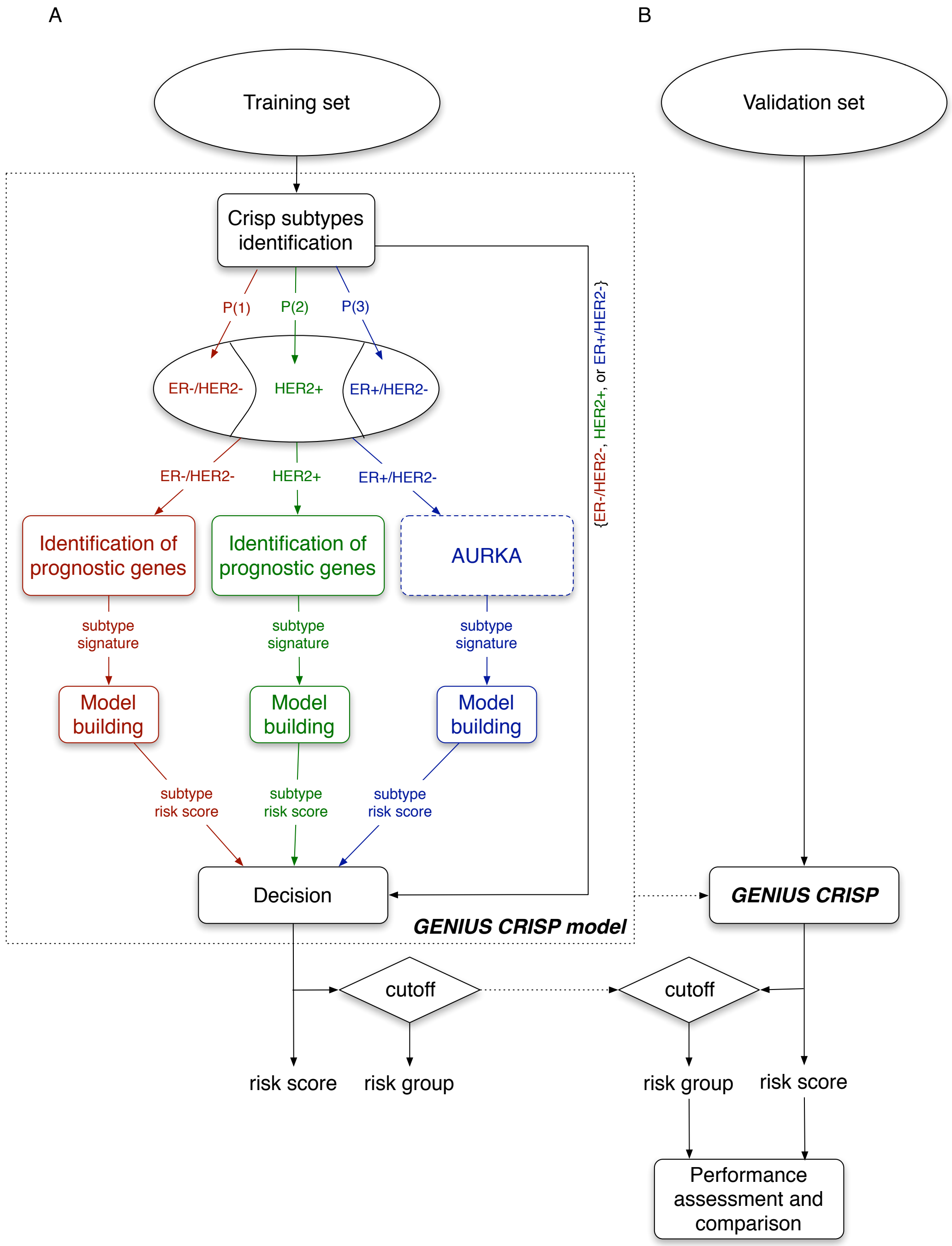

Figure 8: Design of GENIUS CRISP: (a) Training phase to build GENIUS CRISP; (b) Validation phase to test GENIUS CRISP in the independent dataset of untreated breast cancer patients Subtype of a patient is identified with respect to the maximum probability of his tumor belongingness as computed by the subtype clustering model. Only patients having a tumor of a specific subtype are used to identify corresponding subtype signature.

The forestplot is sketched in Figure 18 while the corresponding concordance index esti-

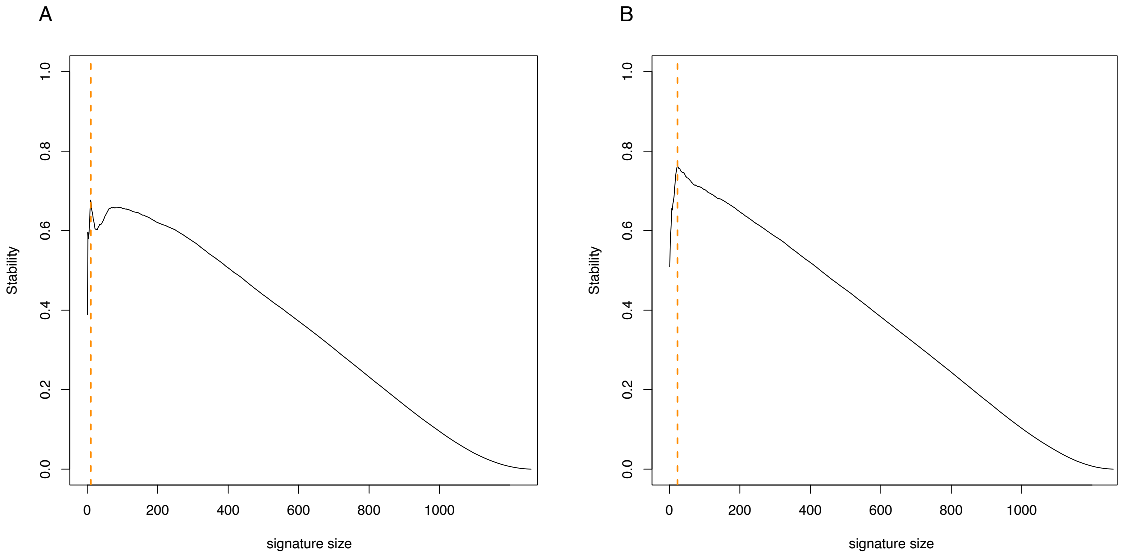

Figure 9: Stability of the subtype signature the (a) ER-/HER2- and (b) HER2+ subtypes respectively.

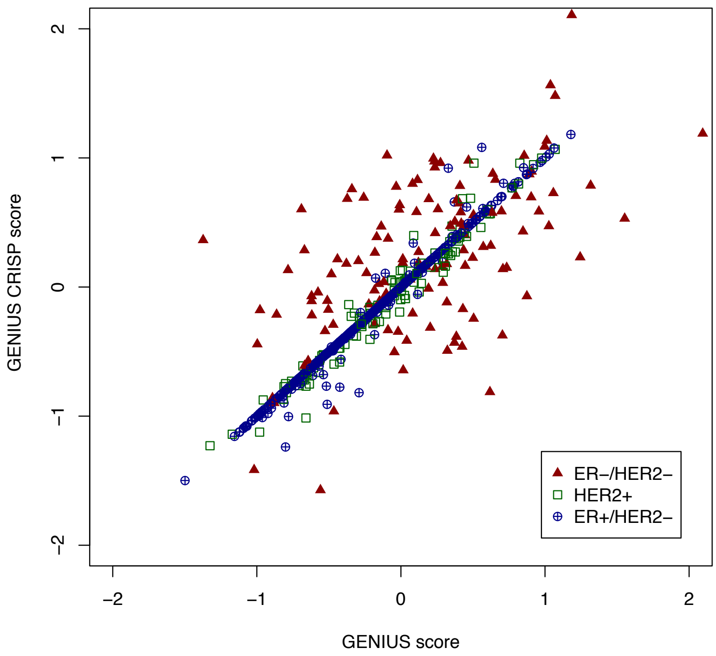

Figure 10: Plot of GENIUS and GENIUS CRISP risk score predictions with respect to the subtypes.

mates, confidence intervals and p-values are given in Table 9. Figure 12 sketches the time-

	Correlation
ER-/HER2-	0.55
HER2+	0.98
ER+/HER2-	0.99
ALL	0.9

Table 8: Correlation between GENIUS and GENIUS CRISP risk score predictions with respect to the subtypes.

dependent ROC curves for GENIUS and GENIUS CRISP with respect to the subtypes.

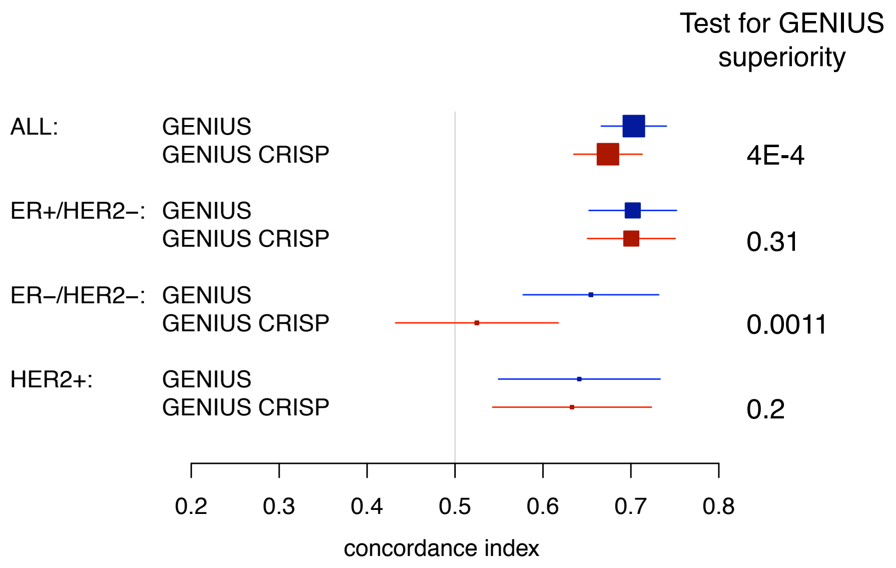

Figure 11: Forestplot of GENIUS and GENIUS CRISP performance for risk score predictions with respect to the subtypes.

5.1.2 Risk Group Predictions

Table 10 reports the discrepancies between GENIUS and GENIUS CRISP risk group predictions with respect to the subtypes.

We observed large discrepancies in the ER-/HER2+ subtypes. In the global population of patients, we observed $\approx$ 8% of patients being classified differently by GENIUS and GENIUS CRISP.

In order to assess whether the differences observed at the risk group predictions level have any impact on prognosis, we compared the performance of GENIUS and GENIUS CRISP. The forestplot is sketched in Figure 13 while the corresponding concordance index estimates, confidence intervals and p-values are given in Table 11.

subtype	classifier	C-index	95%CI	p-value	n
ALL	GENIUS	0.703	[0.666,0.74]	1.0E-27	724
	GENIUS CRISP	0.674	[0.635,0.713]	9.3E-19	724
ER+/HER2-	GENIUS	0.702	[0.652,0.752]	6.9E-16	503
	GENIUS CRISP	0.7	[0.651,0.75]	1.8E-15	503
ER-/HER2-	GENIUS	0.655	[0.577,0.732]	7.1E-05	116
	GENIUS CRISP	0.525	[0.432,0.617]	3.0E-01	116
HER2+	GENIUS	0.641	[0.549,0.733]	9.3E-04	105
	GENIUS CRISP	0.633	[0.543,0.723]	1.9E-03	105

Table 9: Concordance index estimates, confidence intervals, p-values and number of patients for GENIUS and GENIUS CRISP risk score predictions with respect to the subtypes.

subtype	GENIUS CRISP		GENIUS	
ALL			GENIUS	
			0	1
	GENIUS CRISP	0	460	28
		1	28	229
ER+/HER2-			GENIUS	
			0	1
	GENIUS CRISP	0	382	2
		1	9	121
ER-/HER2-			GENIUS	
			0	1
	GENIUS CRISP	0	27	22
		1	15	61
HER2+			GENIUS	
			0	1
	GENIUS CRISP	0	51	4
		1	4	47

Table 10: Contingency table of the GENIUS and GENIUS CRISP risk group predictions.

5.2 Conclusions

The fuzzy version of GENIUS yielded consistently better performance than the crisp one ($p < 0.05$ for GENIUS superiority over GENIUS CRISP in both risk score and risk group predictions in the global population of patients). It is worth to note that the most stable signature

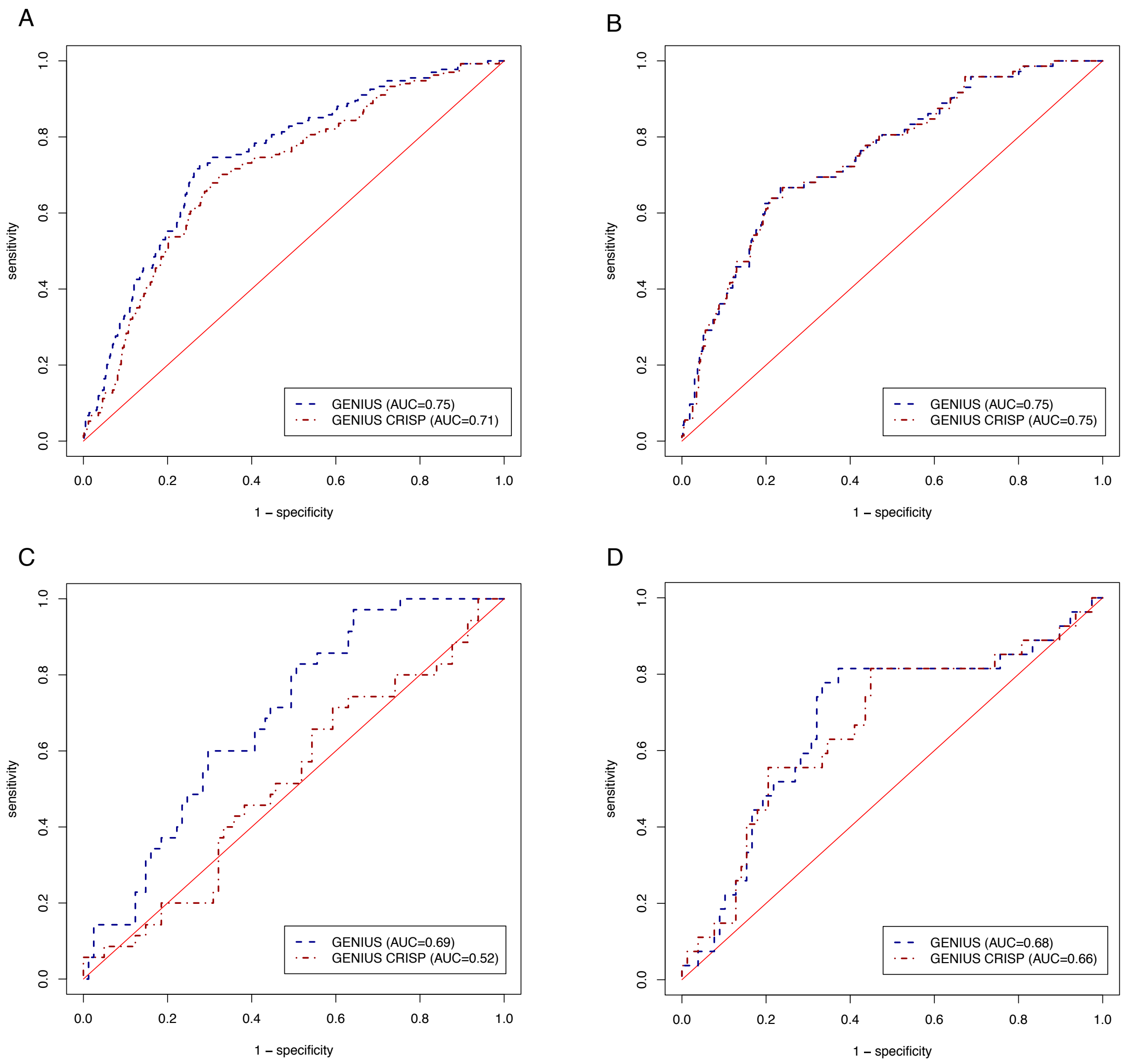

Figure 12: Time-dependent ROC curves for GENIUS and GENIUS CRISP in the (a) global population, the (b) ER+/HER2-, (c) ER-/HER2- and (d) HER2+ subtypes.

for ER-/HER2- subtype in GENIUS CRISP contains only 10 genes and did not yield significant prognostic performance. Increasing the signature size to the second peak of stability (signature size of 91 genes, see Figure 9A) yielded significant prognostic performance in the validation set while staying poorer than the performance of the corresponding GENIUS subtype signature (data not shown).

These results highlight the benefit from a prognostic point of view to take into account the probability of the tumors belonging to each subtype in order to compute accurate risk predictions.

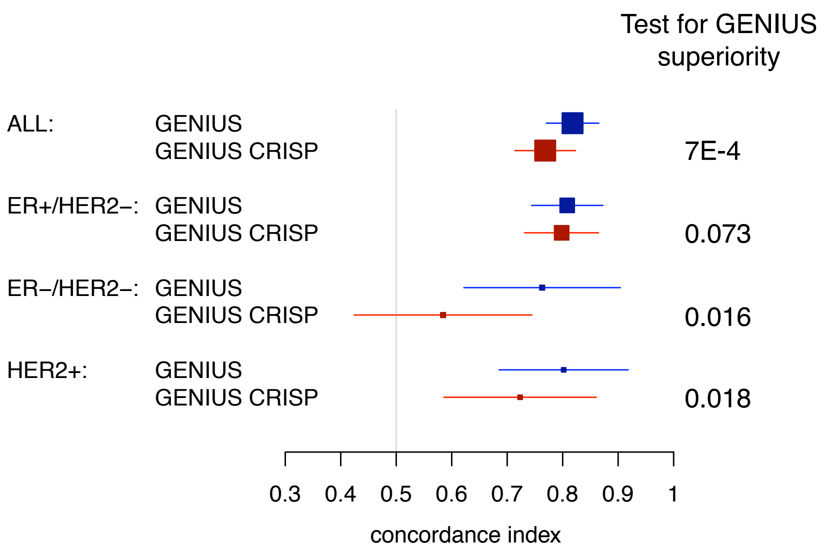

Figure 13: Forestplot of GENIUS and GENIUS CRISP performance for risk group predictions with respect to the subtypes.

subtype	classifier	C-index	95%CI	p-value	n
ALL	GENIUS	0.818	[0.771,0.865]	3.1E-37	724
	GENIUS CRISP	0.769	[0.714,0.823]	3.3E-22	724
ER+/HER2-	GENIUS	0.808	[0.744,0.873]	3.0E-21	503
	GENIUS CRISP	0.798	[0.732,0.865]	8.5E-19	503
ER-/HER2-	GENIUS	0.763	[0.622,0.904]	1.3E-03	116
	GENIUS CRISP	0.584	[0.424,0.745]	1.5E-01	116
HER2+	GENIUS	0.802	[0.685,0.918]	2.9E-07	105
	GENIUS CRISP	0.723	[0.586,0.861]	7.2E-04	105

Table 11: Concordance index estimates, confidence intervals, p-values and number of patients for GENIUS and GENIUS CRISP risk group predictions with respect to the subtypes.

6 Development of SUBCLASSIF and Comparison with GENIUS

In this section, we describe the development of SUBCLASSIF (see Figure 14), a prognostic model using crisp subtypes identification and published gene signatures. This approach consists in a simple integration of the subtypes identification and the current gene signatures in order to perform breast cancer prognostication.

In SUBCLASSIF model, the subtype of each tumor was assigned univocally to the one having the maximum posterior probability estimated during the subtypes identification step (Section 2). We used in this *crisp* risk prediction model, recently published gene signatures known to be prognostic in specific subtypes:

- IRMODULE for patients whose tumor belong to the ER-/HER2- subtype.
- SDPP for patients whose tumor belong to the HER2+ subtype.
- AURKA for patients whose tumor belong to the ER+/HER2- subtype.

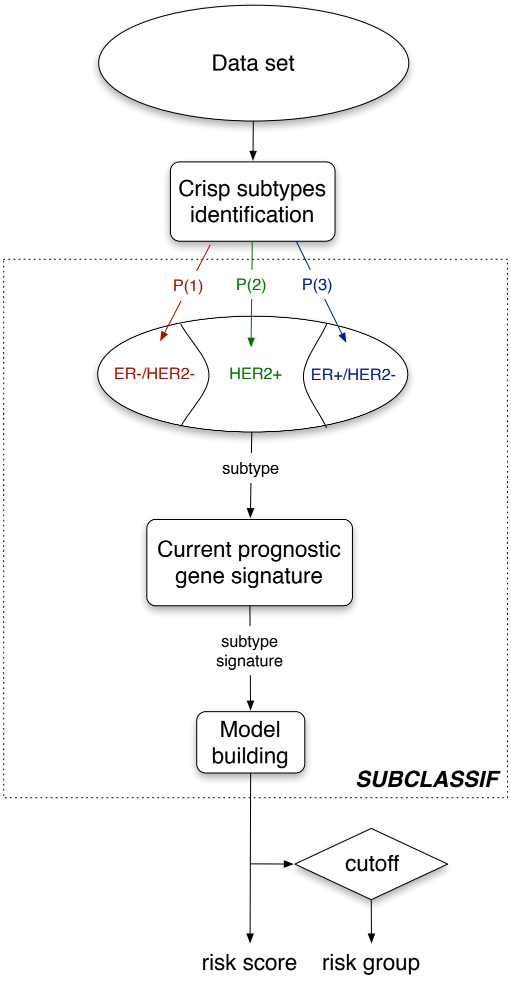

Figure 14: Design of SUBCLASSIF. Current prognostic gene signature is either IRMODULE, SDPP or AURKA for ER-/HER2-, HER2+ or ER+/HER2- subtype respectively.

6.1 GENIUS vs SUBCLASSIF

In order to test whether the approach used for GENIUS outperforms the simple integration of subtypes identification and current prognostic gene signatures used in SUBCLASSIF, we statistically compared these two risk prediction models.

6.1.1 Risk Score Predictions

Figure 15 illustrates the GENIUS and SUBCLASSIF risk score predictions with respect to the subtypes. We observed an overall Pearson's correlation of 0.81.

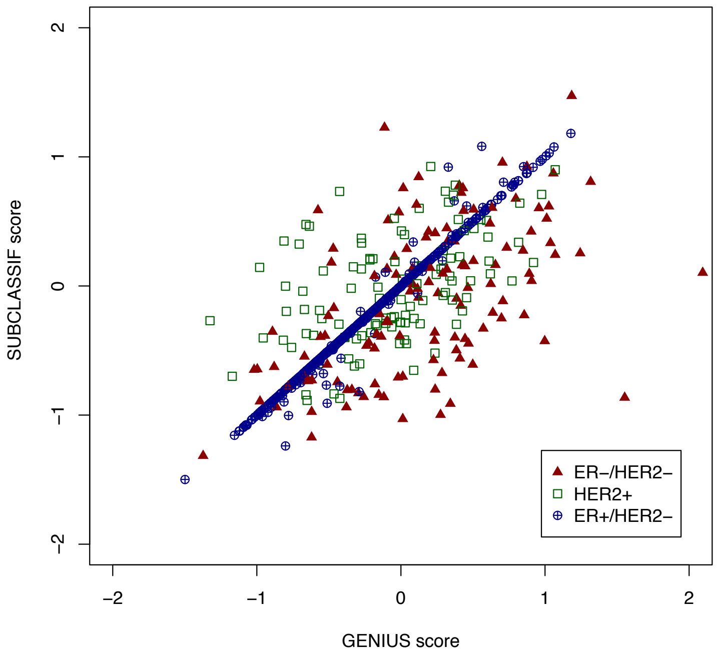

Figure 15: Plot of GENIUS and SUBCLASSIF risk score predictions with respect to the subtypes.

	Correlation
ER-/HER2-	0.53
HER2+	0.48
ER+/HER2-	0.99
ALL	0.81

Table 12: Correlation between GENIUS and SUBCLASSIF risk score predictions with respect to the subtypes.

In terms of risk score predictions, GENIUS yielded better performance whatever the subtypes and significantly outperformed SUBCLASSIF in the global population of patients. Figure 17 sketches the time-dependent ROC curves for GENIUS and SUBCLASSIF. GENIUS

exhibited consistently larger AUC than SUBCLASSIF, with the best improvement observed in the ER-/HER2- subtype. Less clear is the superiority of GENIUS in the HER2+ subtype where SDPP yielded larger sensitivities while GENIUS yielded better specificities when lower sensitivity is allowed.

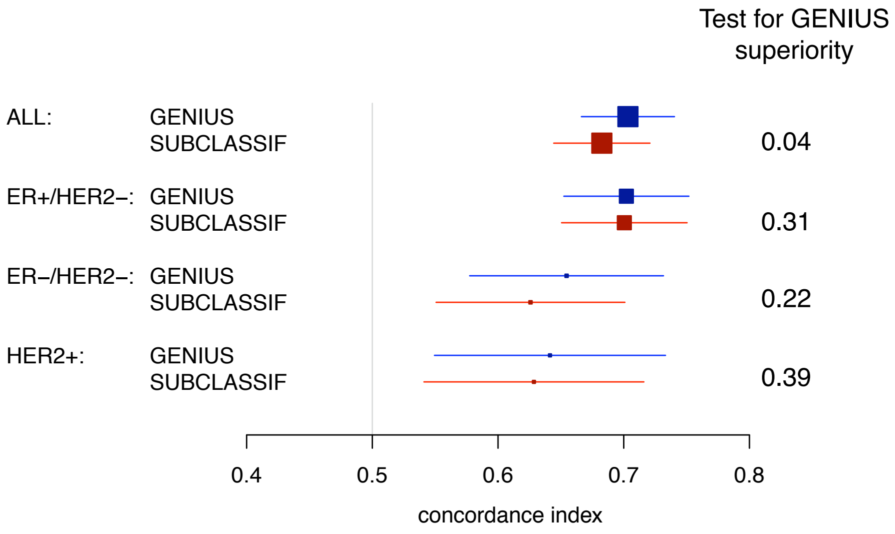

Figure 16: Forestplot of GENIUS and SUBCLASSIF performance for risk score predictions with respect to the subtypes.

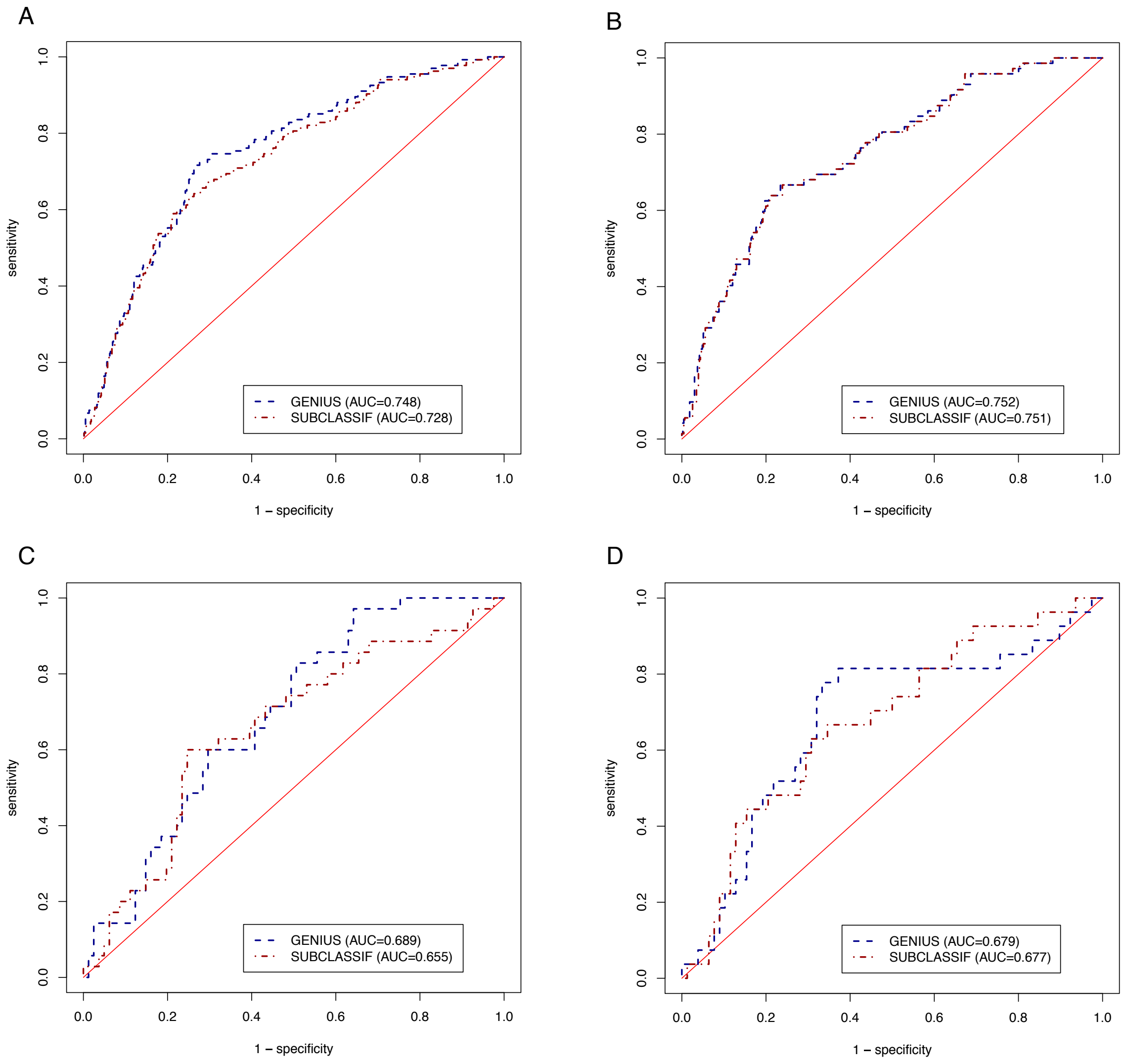

Figure 17: Time-dependent ROC curves for GENIUS and SUBCLASSIF in the (a) global population, the (b) ER+/HER2-, (c) ER-/HER2- and (d) HER2+ subtypes.

6.1.2 Risk Group Predictions

In terms of risk group predictions, GENIUS consistently outperformed SUBCLASSIF, its superiority being significant in the HER+ subtype and in the global population of patients.

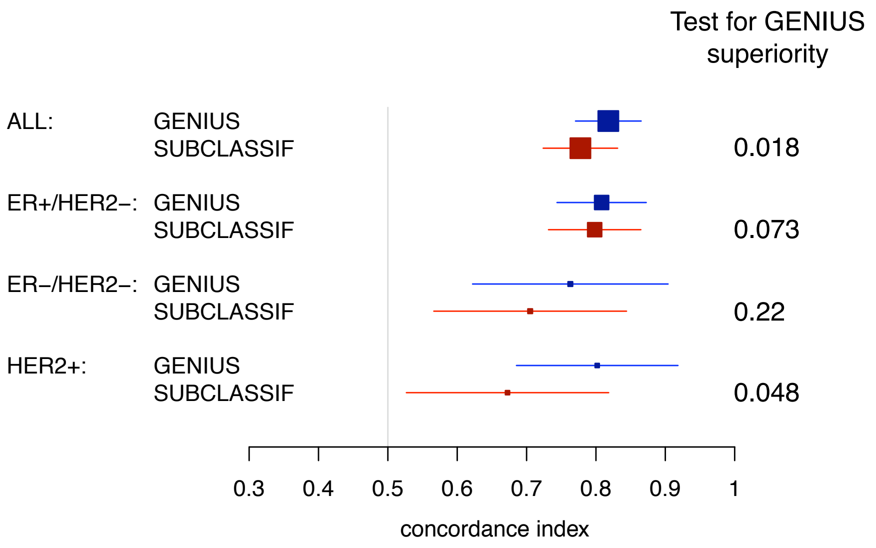

Figure 18: Forestplot of GENIUS and SUBCLASSIF performance for risk group predictions with respect to the subtypes.

6.2 Conclusions

We observed that GENIUS model significantly outperformed the simple approach used in SUBCLASSIF both at the level of risk score and risk group predictions.

7 Supplementary Figures

Supplementary Figure 1

Supplementary Figure 1 sketches the datasets used for each analysis: (a) subtype identification; (b) training set used to build GENIUS; (c) validation set used to assess the performance of GENIUS; (d) validation set to compare the performance of GENIUS vs the state-of-the-art prognostic signatures and the clinical prognostic indices.

Note that there are a few samples in common between UPP, UNT and TBG datasets. We therefore removed from UNT the common patients in TBG and from UPP the common patients in TBG and UNT.

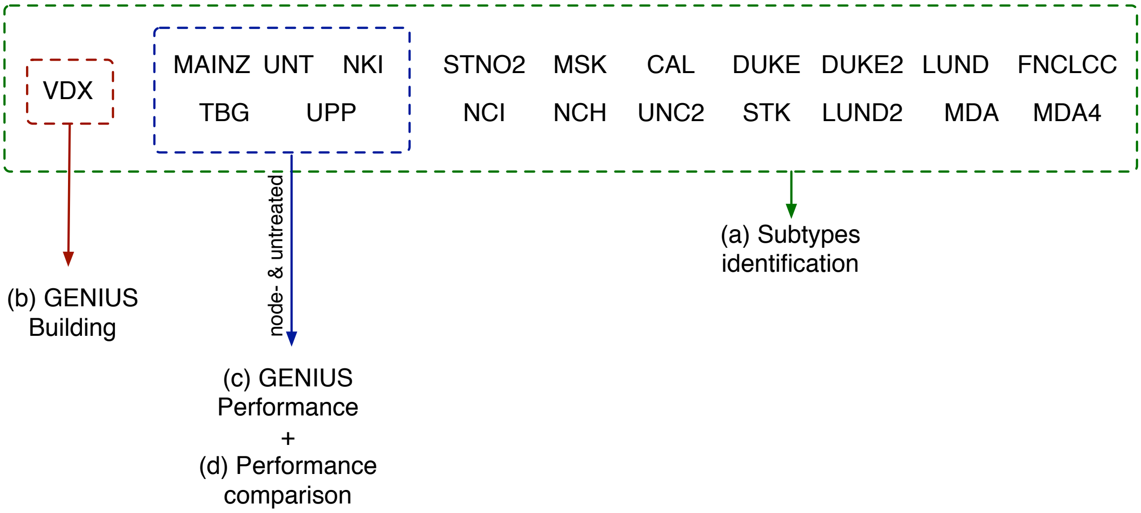

Supplementary Figure 2

Supplementary Figure 2 sketches the subtype signature stability with respect to the signature size for the (a) ER-/HER2- and (b) HER2+ subtypes. The number of genes to include in the subtype signature (orange dashed line) was selected in order to maximize the stability with respect to the size by sampling 90% of the training set 200 times.

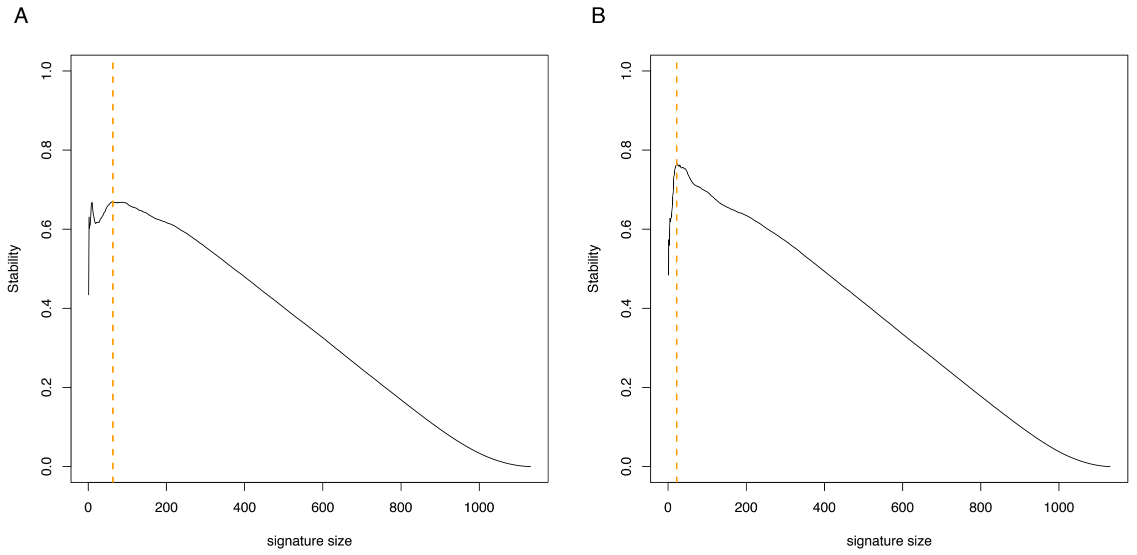

Supplementary Figure 3

Supplementary Figure 3 reports the correlations between GENIUS and the other prognostic gene signatures as well as the clinical prognostic indices.

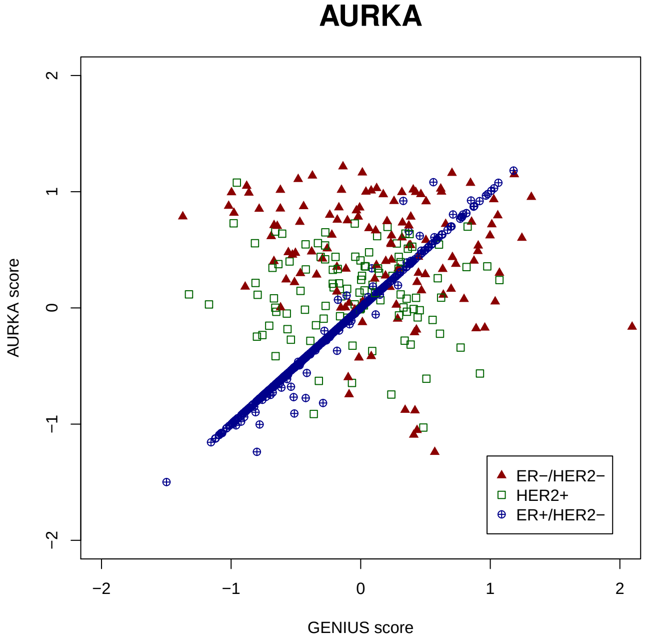

	Correlation
ER-/HER2-	-0.21
HER2+	-0.078
ER+/HER2-	0.99
ALL	0.64

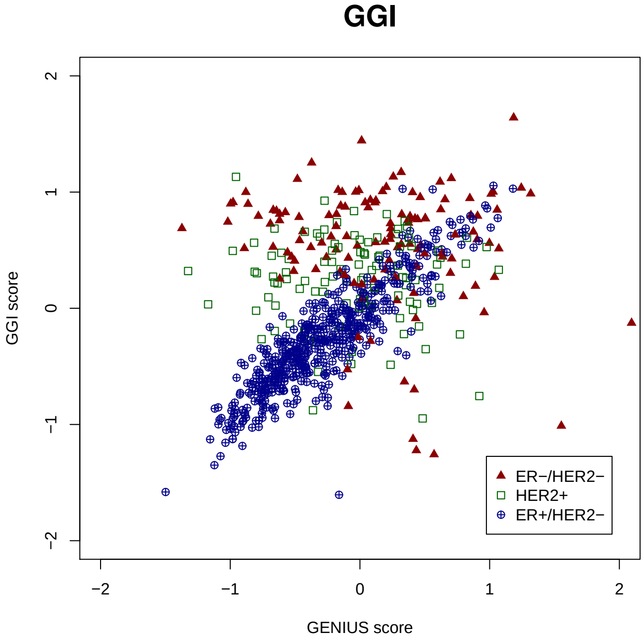

	Correlation
ER-/HER2-	-0.15
HER2+	-0.068
ER+/HER2-	0.89
ALL	0.6

STAT1

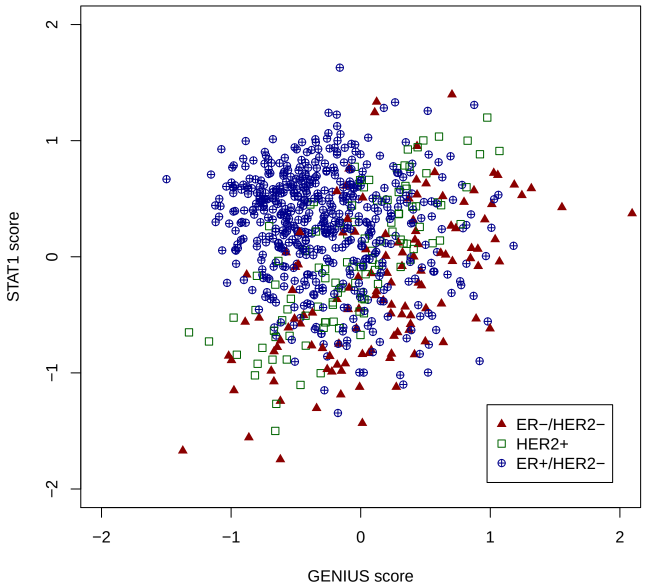

	Correlation
ER-/HER2-	0.55
HER2+	0.75
ER+/HER2-	-0.16
ALL	0.027

PLAU

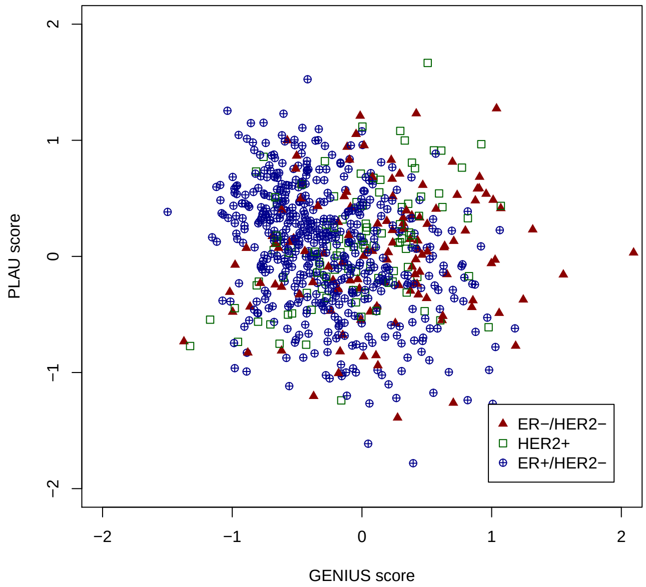

	Correlation
ER-/HER2-	0.11
HER2+	0.37
ER+/HER2-	-0.35
ALL	-0.15

IRMODULE

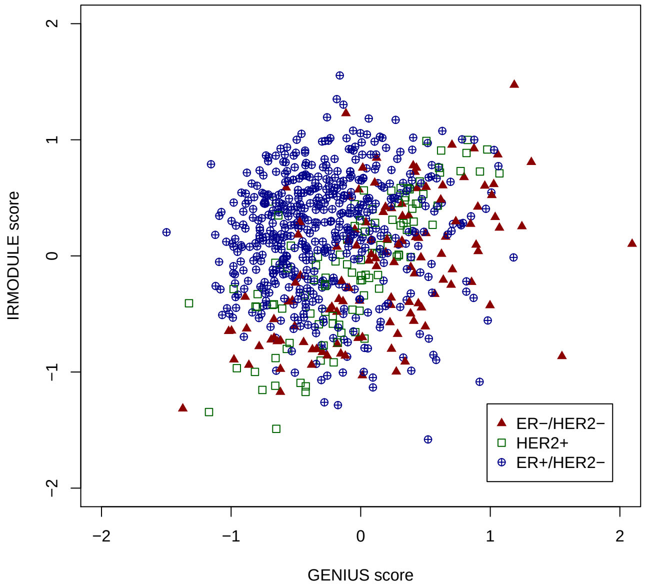

	Correlation
ER-/HER2-	0.53
HER2+	0.76
ER+/HER2-	0.086
ALL	0.19

SDPP

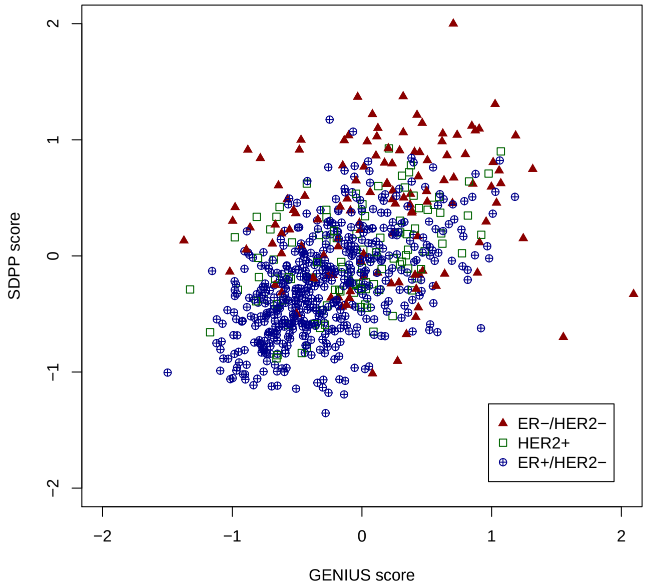

	Correlation
ER-/HER2-	0.18
HER2+	0.48
ER+/HER2-	0.5
ALL	0.5

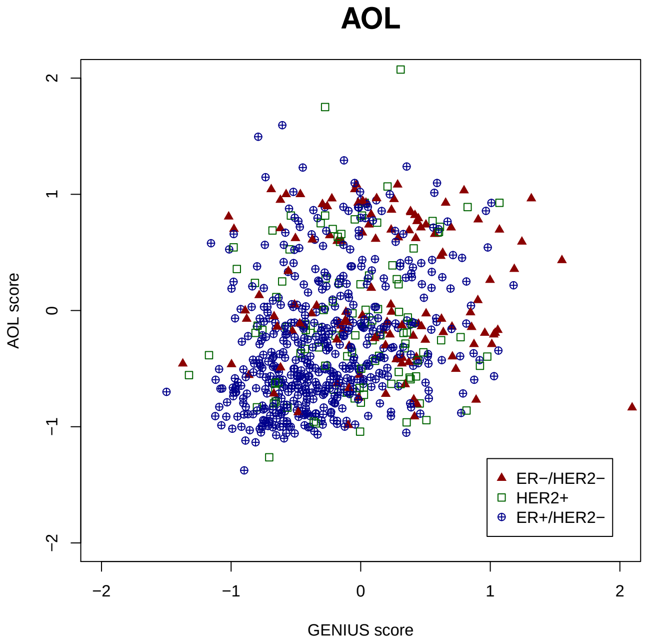

	Correlation
ER-/HER2-	-0.026
HER2+	0.093
ER+/HER2-	0.3
ALL	0.27

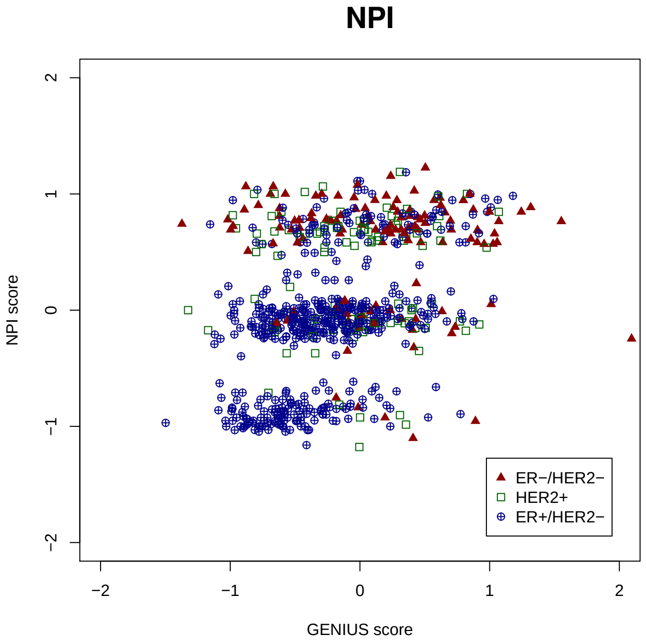

	Correlation
ER-/HER2-	-0.099
HER2+	0.011
ER+/HER2-	0.46
ALL	0.38

Supplementary Figure 4

Supplementary Figure 4 sketches the time-dependent ROC curves for GENIUS and all the state-of-the-art prognostic gene signatures in the (a) global population, the (b) ER+/HER2-, (c) ER-/HER2- and (d) HER2+ subtypes.

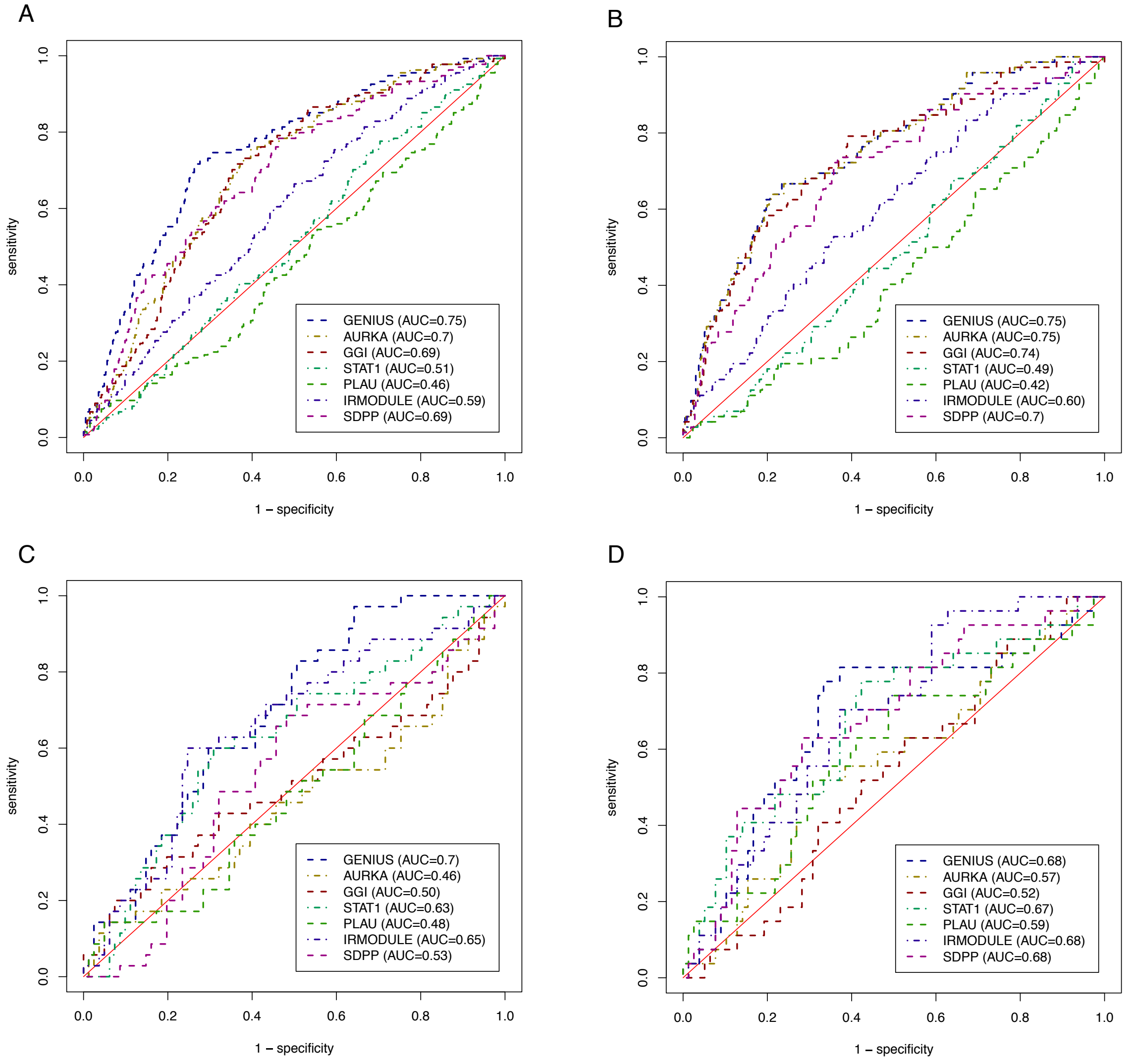

Supplementary Figure 5

Supplementary Figure 5 sketches the time-dependent ROC curves at 5 years for the risk score predictions computed by GENIUS, GGI, AOL and NPI, in the (a) global population, the (b) ER+/HER2-, (c) ER-/HER2- and (d) HER2+ subtypes in the validation set.

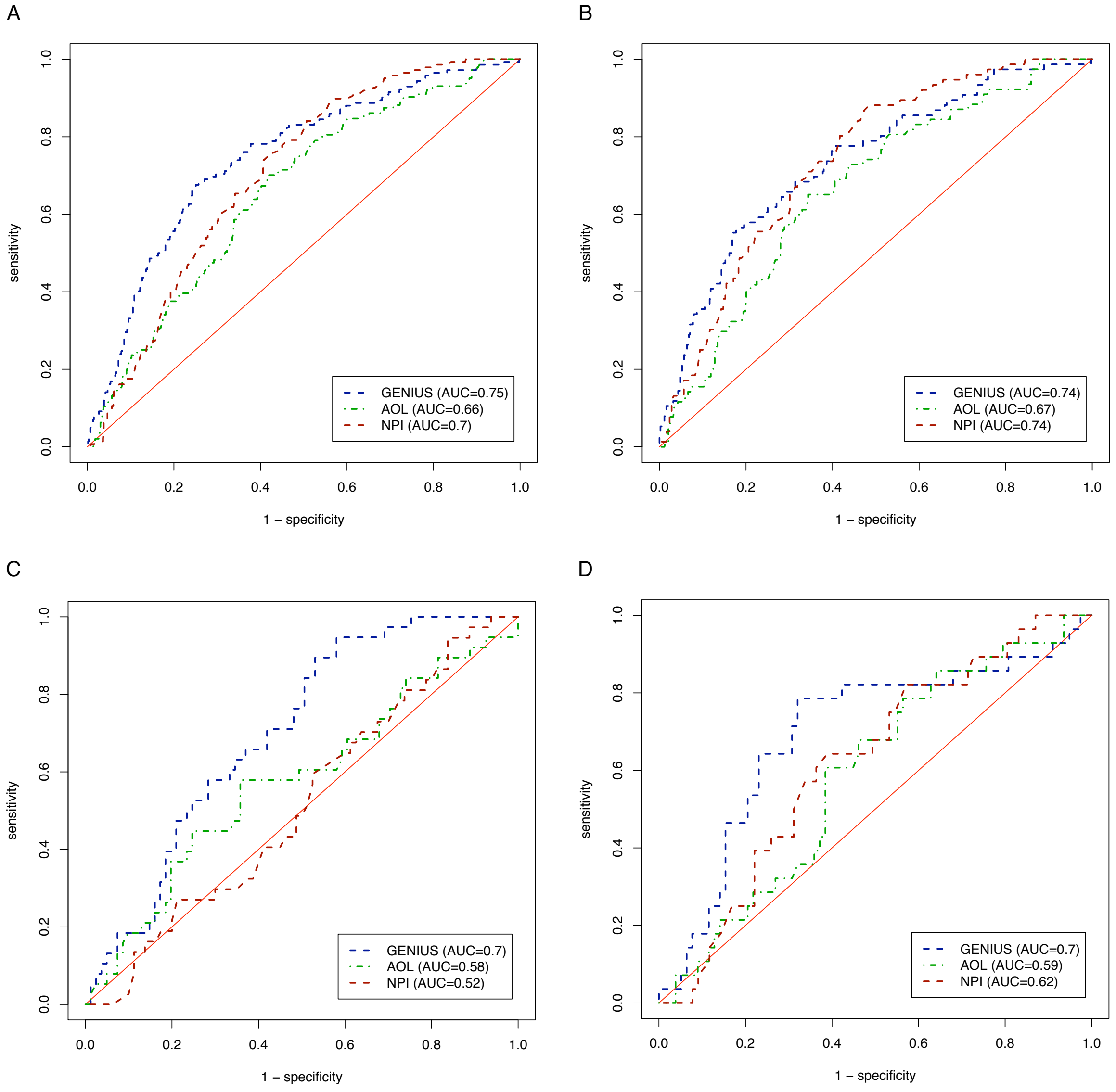

Supplementary Figure 6

Supplementary Figure 6 sketches the Kaplan-Meier survival curves for AOL and NPI risk group predictions in the (AOL: a, NPI: b) global population, the (AOL: c, NPI: d) ER+/HER2-, (AOL: e, NPI: f) ER-/HER2- and (AOL: g, NPI: h) HER2+ subtypes in the validation set.

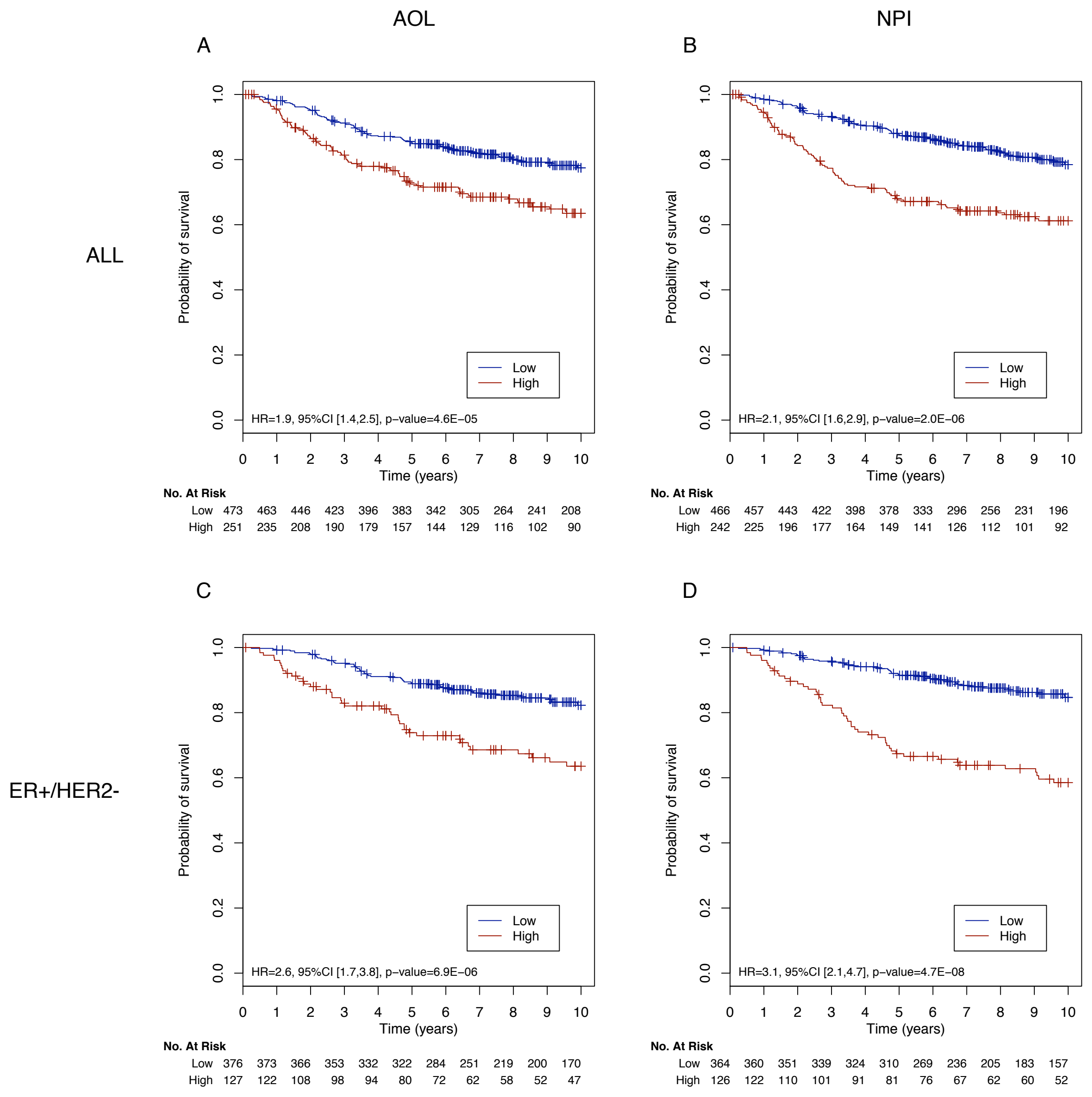

ER-/HER2-

E

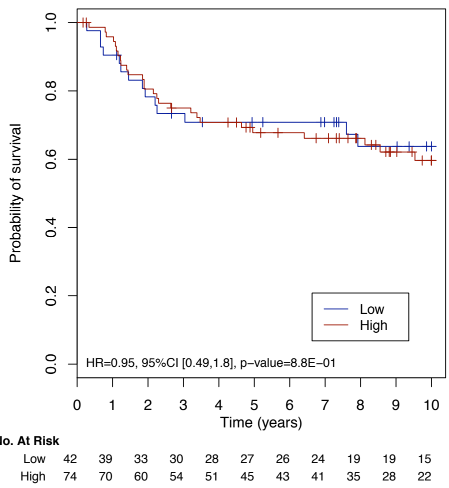

F

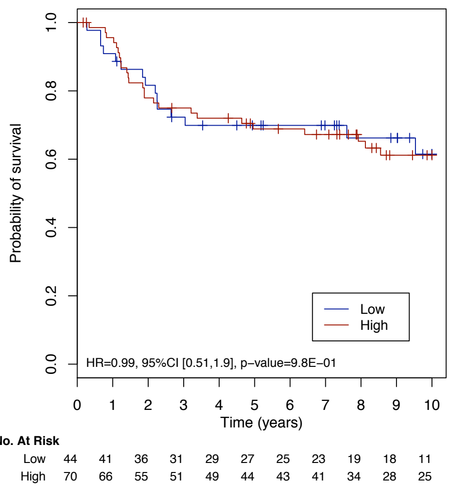

HER2+

G

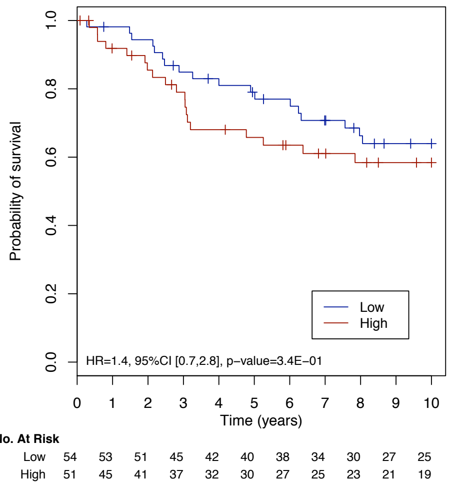

H

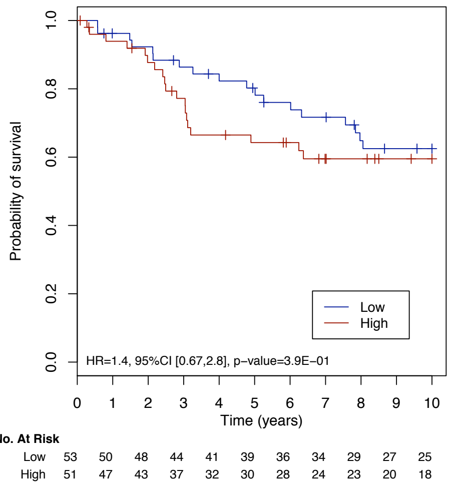

Supplementary Figure 7

Supplementary Figure 7 sketches the Kaplan-Meier survival curves for the combination of GENIUS and AOL/NPI predictions using the official cutoffs[4] in the (AOL: a; NPI: b) global population, the (AOL: c; NPI: d) ER+/HER2-, (AOL: e; AOL: f) ER-/HER2- and (AOL: g; NPI: h) HER2+ subtypes of the validation set.

ALL

A

GENIUS/AOL

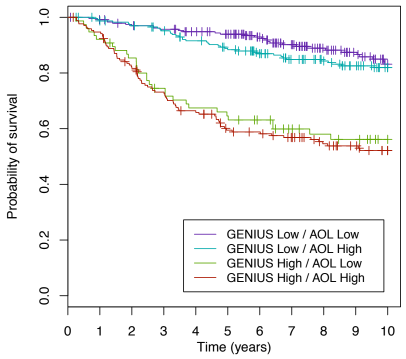

No. At Risk

GENIUS Low / AOL Low	237	233	226	222	214	206	176	153	125	110	91
GENIUS Low / AOL High	233	227	222	214	203	193	176	161	149	136	123
GENIUS High / AOL Low	76	71	64	54	48	46	42	35	32	30	23
GENIUS High / AOL High	171	162	137	121	108	93	90	83	73	67	58

B

GENIUS/NPI

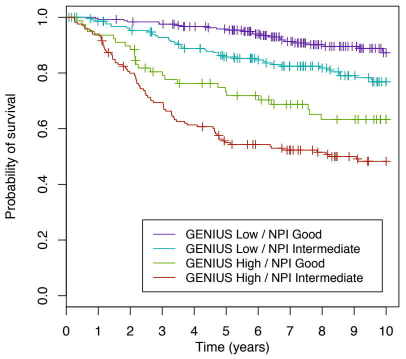

No. At Risk

GENIUS Low / NPI Good	243	242	239	236	227	220	192	167	144	129	110
GENIUS Low / NPI Intermediate	216	208	199	189	179	168	153	139	123	111	98
GENIUS High / NPI Good	79	74	69	59	55	51	48	42	36	35	26
GENIUS High / NPI Intermediate	166	157	130	113	99	86	81	75	68	60	54

ER+/HER2-

C

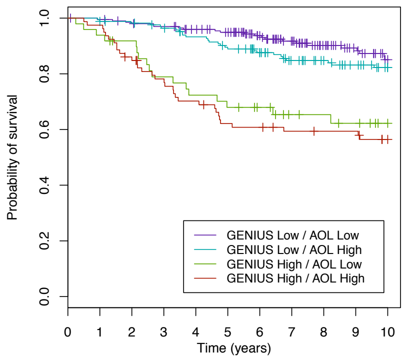

No. At Risk

GENIUS Low / AOL Low	202	201	196	193	185	178	149	128	105	92	76
GENIUS Low / AOL High	168	166	164	159	151	143	130	117	107	98	88
GENIUS High / AOL Low	49	47	44	37	34	32	29	23	22	20	14
GENIUS High / AOL High	79	78	67	60	54	47	46	43	42	42	36

D

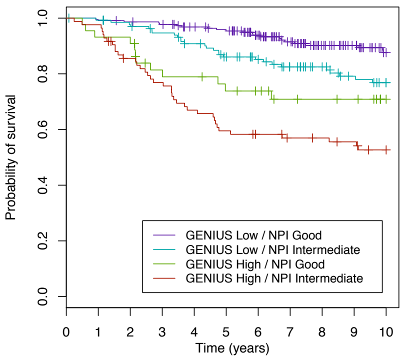

No. At Risk

GENIUS Low / NPI Good	223	222	220	217	208	202	174	150	128	115	96
GENIUS Low / NPI Intermediate	135	134	129	124	117	108	98	87	77	69	62
GENIUS High / NPI Good	44	42	41	34	33	30	28	24	23	22	16
GENIUS High / NPI Intermediate	84	83	70	63	55	49	46	43	42	40	35

[4] Official cutoff for NPI (Good, Intermediate, Poor) and the cutoff for AOL that had been suggested in the TRANSBIG validation studies.

E

F

ER-/HER2-

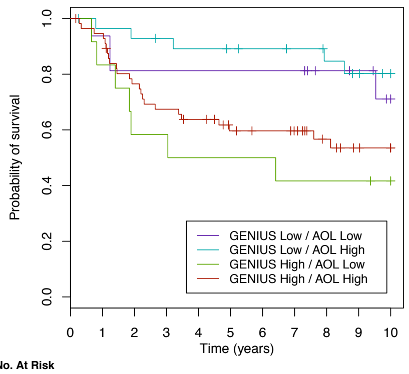

No. At Risk	0	1	2	3	4	5	6	7	8	9	10
GENIUS Low / AOL Low	16	16	15	15	15	15	15	15	11	10	6
GENIUS Low / AOL High	29	28	27	26	25	24	23	22	20	18	15
GENIUS High / AOL Low	12	11	8	8	7	7	7	6	6	6	4
GENIUS High / AOL High	57	54	43	38	35	29	27	25	19	15	12

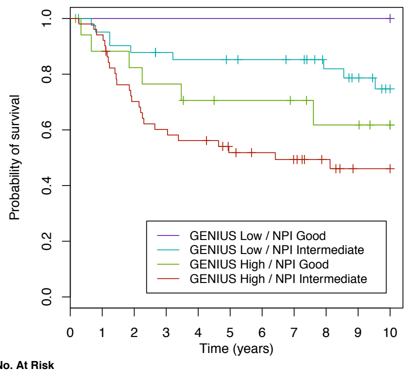

No. At Risk	0	1	2	3	4	5	6	7	8	9	10
GENIUS Low / NPI Good	4	4	4	4	4	4	4	4	4	4	4
GENIUS Low / NPI Intermediate	42	40	37	35	34	33	32	31	26	23	17
GENIUS High / NPI Good	18	16	15	14	12	11	11	10	8	8	4
GENIUS High / NPI Intermediate	51	49	36	31	29	24	22	20	16	12	11

G

H

HER2+

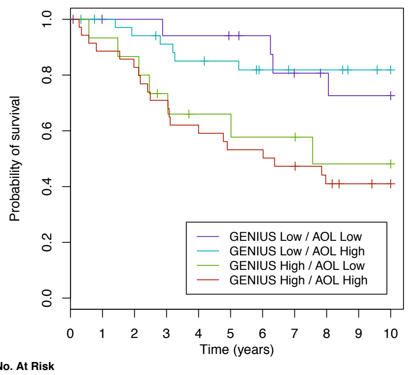

No. At Risk	0	1	2	3	4	5	6	7	8	9	10
GENIUS Low / AOL Low	19	18	18	17	17	16	15	12	11	10	9
GENIUS Low / AOL High	36	35	33	31	29	28	25	24	24	22	20
GENIUS High / AOL Low	15	15	14	11	9	9	8	8	6	6	5
GENIUS High / AOL High	35	32	29	25	21	19	19	17	14	12	10

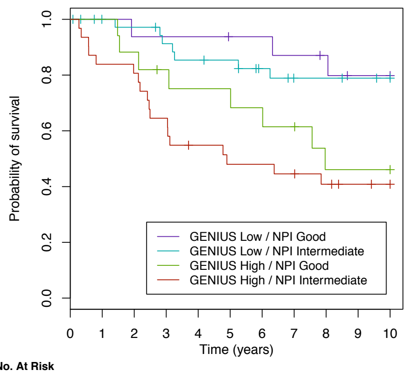

No. At Risk	0	1	2	3	4	5	6	7	8	9	10
GENIUS Low / NPI Good	16	16	16	16	16	15	15	14	13	11	10
GENIUS Low / NPI Intermediate	39	36	35	32	30	29	25	22	22	21	19
GENIUS High / NPI Good	17	17	16	13	12	12	11	10	7	7	6
GENIUS High / NPI Intermediate	31	27	26	21	17	15	15	14	12	10	8

8 Supplementary Tables

Supplementary Table 1

Supplementary Table 1 describes all the cohorts of breast cancer patients in terms of clinical information.

Reference	Dataset	Technology	Survival	Treatment	Patients	Probes
[25,64]	NKI	Agilent	RFS, DMFS, OS	untreated, chemo	345	24,481
[5]	STNO2	cDNA Stanford	RFS, OS	untreated, chemo, hormono	122	7,787
[6]	NCI	cDNA NCI	RFS	untreated, chemo, hormono	99	6,878
[61]	MSK	Affymetrix	DMFS	heterogeneous	99	22,283
[41]	UPP	Affymetrix	RFS	untreated, hormono	251	22,283
[65]	STK	Affymetrix	RFS	untreated, chemo, hormono	159	22,283
[18,19]	VDX	Affymetrix	RFS, DMFS	untreated	344	22,283
[24]	UNT	Affymetrix	RFS, DMFS	untreated	137	22,283
[66]	UNC2	Agilent	RFS, OS	heterogeneous	248	21,495
[53]	DUKE	Affymetrix	OS	heterogeneous	171	12,625
[52]	CAL	Affymetrix	RFS, DMFS, OS	chemo, hormono	118	22,283
[34]	TBG	Affymetrix	RFS, DMFS, OS	untreated	198	22,283
[63]	NCH	Agilent	RFS, DMFS, OS	untreated, chemo, hormono	135	17,086
[54]	DUKE2	Affymetrix	NA	chemo	160	61,359
[58]	MAINZ	Affymetrix	DMFS	untreated	200	22,283
[57]	LUND2	Swegene	NA	hormono	105	27,648
[56]	LUND	Swegene	NA	heterogeneous	143	26,824
[55]	FNCLCC	Nylon FNCLCC	NA	chemo	150	9,216
[59]	MDA	Affymetrix	NA	chemo	133	22,283
[60]	MDA4	Affymetrix	NA	chemo	129	22,283

Supplementary Table 2

Supplementary Table 2 gives the subtype identification and probabilities to belong to each subtypes as computed by the clustering model on the 20 public breast cancer microarray datasets used in this study.

Columns description :

samplename Identifier of the tumor sample.

dataset Identifier of the dataset including the tumor sample.

proba.ER-/HER2- Posterior probability for the sample to belong the the ER-/HER2- molecular subtype.

proba.HER2+ Posterior probability for the sample to belong the the HER2+ molecular subtype.

proba.ER+/HER2- Posterior probability for the sample to belong the the ER+/HER2- molecular subtype.

most.likely.subtype Molecular subtype of the tumor sample wit the maximum posterior probability of benlonging.

Supplementary Table 3

Supplementary Table 3 lists the genes selected for the subtype signatures (ER-/HER2-, HER2+ and ER+/HER2-).

Columns description :

subtype.signature Name of the molecular subtype for which the prognostic genes (subtype signature) were selected.

probe Identifier of the Affymetrix probe set.

EntrezGene.ID Entrez gene id as defined by the Entrez Gene database[a].

coefficient Coefficient $\{-1, +1\}$ used to compute the subtype risk score.

NCBI.gene.symbol NCBI gene symbol as defined by the Entrez Gene database.

Description Description of the gene.

[a] http://www.ncbi.nlm.nih.gov/sites/entrez?db=gene

Supplementary Table 4

Supplementary Table 4 reports the C-index estimates, confidence intervals and p-values for the risk score predictions of GENIUS, all the state-of-the-art prognostic signatures and clinical prognostic indices.

Subtype	Signature	C-index	95%CI	p-value	n
	GENIUS	0.7	[0.67,0.74]	2.6E-27	724
	AURKA	0.67	[0.63,0.71]	4.5E-19	724
	GGI	0.67	[0.63,0.71]	9.1E-19	724
	STAT1	0.51	[0.47,0.55]	3.0E-01	724
ALL	PLAU	0.47	[0.42,0.51]	7.8E-02	724
	IRMODULE	0.58	[0.54,0.62]	5.1E-05	724
	SDPP	0.66	[0.62,0.7]	2.6E-16	724
	AOL	0.63	[0.59,0.67]	2.5E-11	724
	NPI	0.67	[0.63,0.7]	3.2E-18	708
	GENIUS	0.7	[0.65,0.75]	8.4E-16	503
	AURKA	0.7	[0.65,0.75]	1.8E-15	503
	GGI	0.7	[0.64,0.75]	4.3E-14	503
	STAT1	0.51	[0.46,0.57]	3.5E-01	503
ER+/HER2-	PLAU	0.44	[0.38,0.5]	2.0E-02	503
	IRMODULE	0.6	[0.54,0.65]	2.0E-04	503
	SDPP	0.67	[0.62,0.72]	1.4E-10	503
	AOL	0.65	[0.59,0.7]	3.7E-08	503
	NPI	0.69	[0.64,0.74]	4.9E-14	490
	GENIUS	0.65	[0.57,0.73]	7.1E-05	116
	AURKA	0.47	[0.38,0.57]	3.0E-01	116
	GGI	0.51	[0.41,0.6]	4.3E-01	116
	STAT1	0.6	[0.52,0.68]	5.1E-03	116
ER-/HER2-	PLAU	0.49	[0.4,0.58]	4.3E-01	116
	IRMODULE	0.63	[0.55,0.70]	5.1E-04	116
	SDPP	0.55	[0.46,0.64]	1.4E-01	116
	AOL	0.54	[0.44,0.63]	2.2E-01	116
	NPI	0.52	[0.43,0.62]	3.1E-01	114
	GENIUS	0.65	[0.55,0.74]	9.3E-04	105
	AURKA	0.56	[0.46,0.65]	1.2E-01	105
	GGI	0.52	[0.43,0.61]	2.9E-01	105
	STAT1	0.61	[0.53,0.7]	4.9E-03	105
HER2+	PLAU	0.58	[0.49,0.68]	4.9E-02	105
	IRMODULE	0.65	[0.57,0.73]	9.5E-05	105
	SDPP	0.63	[0.55,0.72]	1.4E-03	105
	AOL	0.56	[0.48,0.64]	6.3E-02	105
	NPI	0.56	[0.48,0.65]	7.7E-02	104

Supplementary Table 5

Supplementary Table 5 reports the proportions of patients in the low- and high-risk groups with respect to the subtypes as computed by GENIUS and GGI in the validation set. Note that GGI classified most of ER-/HER2- patients at high risk (86%) in contrast to GENIUS (60%).

Subtype	GENIUS		GGI		Total
	Low-risk	High-risk	Low-risk	High-risk	
ER-/HER2-	50 (40%)	75 (60%)	17 (14%)	108 (86%)	125
HER2+	50 (47%)	56 (52%)	37 (35%)	69 (65%)	106
ER+/HER2-	384 (74%)	130 (25%)	430 (84%)	84 (16%)	514
ALL	484 (64%)	261 (35%)	484 (65%)	261 (35%)	745

Supplementary Table 6

Supplementary Table 6 reports the C-index estimates, confidence intervals and p-values for the risk group predictions of GENIUS, all the state-of-the-art prognostic signatures and clinical prognostic indices.

Subtype	Signature	C-index	95%CI	p-value	n
ALL	GENIUS	0.82	[0.77,0.86]	6.8E-40	724
	AURKA	0.72	[0.65,0.78]	4.3E-12	724
	GGI	0.72	[0.65,0.78]	3.1E-12	724
	STAT1	0.5	[0.42,0.58]	4.9E-01	724
	PLAU	0.4	[0.32,0.48]	9.1E-03	724
	IRMODULE	0.59	[0.51,0.66]	9.6E-03	724
	SDPP	0.73	[0.67,0.79]	1.3E-13	724
	AOL	0.66	[0.59,0.73]	2.5E-06	724
	NPI	0.72	[0.66,0.78]	2.2E-12	708
ER+/HER2-	GENIUS	0.81	[0.74,0.87]	3.0E-21	503
	AURKA	0.8	[0.73,0.86]	8.5E-19	503
	GGI	0.78	[0.71,0.85]	2.1E-15	503
	STAT1	0.46	[0.34,0.58]	2.8E-01	503
	PLAU	0.43	[0.32,0.55]	1.2E-01	503
	IRMODULE	0.63	[0.54,0.73]	3.3E-03	503
	SDPP	0.73	[0.65,0.81]	2.7E-08	503
	AOL	0.72	[0.64,0.8]	1.4E-07	503
	NPI	0.79	[0.72,0.86]	6.8E-17	490
ER-/HER2-	GENIUS	0.76	[0.62,0.90]	1.3E-04	116
	AURKA	0.39	[0.24,0.54]	6.9E-02	116
	GGI	0.49	[0.33,0.64]	4.3E-01	116
	STAT1	0.71	[0.57,0.85]	1.9E-03	116
	PLAU	0.45	[0.29,0.6]	2.4E-01	116
	IRMODULE	0.70	[0.57,0.84]	1.9E-03	116
	SDPP	0.62	[0.46,0.77]	7.1E-02	116
	AOL	0.5	[0.34,0.67]	4.8E-01	116
	NPI	0.5	[0.34,0.66]	4.9E-01	114
HER2+	GENIUS	0.80	[0.68,0.92]	1.9E-07	105
	AURKA	0.54	[0.38,0.7]	3.1E-01	105
	GGI	0.53	[0.37,0.69]	3.5E-01	105
	STAT1	0.74	[0.61,0.88]	2.8E-04	105
	PLAU	0.63	[0.48,0.79]	4.6E-02	105
	IRMODULE	0.76	[0.63,0.89]	4.7E-05	105
	SDPP	0.68	[0.54,0.83]	7.3E-03	105
	AOL	0.59	[0.44,0.75]	1.2E-01	105
	NPI	0.59	[0.43,0.74]	1.4E-01	104
